# Cell Wall Degrading Enzyme Induced Rice Innate Immune Responses Are Suppressed by the Type 3 Secretion System Effectors XopN, XopQ, XopX and XopZ of *Xanthomonas* *oryzae* pv. *oryzae*

**DOI:** 10.1371/journal.pone.0075867

**Published:** 2013-09-26

**Authors:** Dipanwita Sinha, Mahesh Kumar Gupta, Hitendra Kumar Patel, Ashish Ranjan, Ramesh V. Sonti

**Affiliations:** CSIR-Centre for Cellular and Molecular Biology, Hyderabad, Andhra Pradesh, India; Universidad de Costa Rica, Costa Rica

## Abstract

Innate immune responses are induced in plants and animals through perception of Damage Associated Molecular Patterns. These immune responses are suppressed by pathogens during infection. A number of studies have focussed on identifying functions of plant pathogenic bacteria that are involved in suppression of Pathogen Associated Molecular Pattern induced immune responses. In comparison, there is very little information on functions used by plant pathogens to suppress Damage Associated Molecular Pattern induced immune responses. *Xanthomonas* *oryzae* pv. *oryzae* , a gram negative bacterial pathogen of rice, secretes hydrolytic enzymes such as LipA (Lipase/Esterase) that damage rice cell walls and induce innate immune responses. Here, we show that *Agrobacterium* mediated transient transfer of the gene for XopN, a *X* *. oryzae* pv. *oryzae* type 3 secretion (T3S) system effector, results in suppression of rice innate immune responses induced by LipA. A *xopN* ^*-*^ mutant of *X* *. oryzae* pv. *oryzae* retains the ability to suppress these innate immune responses indicating the presence of other functionally redundant proteins. In transient transfer assays, we have assessed the ability of 15 other *X* *. oryzae* pv. *oryzae* T3S secreted effectors to suppress rice innate immune responses. Amongst these proteins, XopQ, XopX and XopZ are suppressors of LipA induced innate immune responses. A mutation in any one of the *xopN, xopQ*, *xopX* or *xopZ* genes causes partial virulence deficiency while a *xopN* ^*-*^ *xopX* ^-^ double mutant exhibits a greater virulence deficiency. A *xopN* ^*-*^ *xopQ* ^*-*^ *xopX* ^*-*^ *xopZ* ^*-*^ quadruple mutant of *X* *. oryzae* pv. *oryzae* induces callose deposition, an innate immune response, similar to a *X* *. oryzae* pv. *oryzae* T3S^-^ mutant in rice leaves. Overall, these results indicate that multiple T3S secreted proteins of *X* *. oryzae* pv. *oryzae* can suppress cell wall damage induced rice innate immune responses.

## Introduction

The innate immune systems of plants and animals are activated by the perception of danger signals in the form of pathogen associated molecular patterns (PAMPs) and damage-associated molecular patterns (DAMPs). PAMPs are indispensable, structurally conserved molecular features that are unique to a broad class of microbes and typify ‘non-self’ because they are not present in the host [1] whereas DAMPs are mostly endogenous molecules which are released upon tissue injury that occurs during growth, stress and pathogen entry [2]. Pattern recognition receptors (PRRs) are involved in perception of PAMPs and DAMPs. In animals, recognition of either PAMPs or DAMPs activates the innate immune system and results in various inflammatory responses [3]. In plants, the perception of these danger signals results in the activation of the first layer of the plant innate immune system which is termed as PAMP-triggered immunity or PTI [4,5]. Suppression of PTI appears to be a crucial attribute of plant pathogens. A number of studies have shown that Gram negative plant pathogenic bacteria suppress PTI using proteins that are secreted into plant cells via the type 3 secretion system (T3S).

The gram negative bacterial genus *Xanthomonas* is comprised of bacteria that cause almost ~ 400 different plant diseases. These bacteria secrete a number of effector proteins into their host cells using the T3S. These include both TAL (transcription activator-like) effectors and non-TAL effectors. Unlike TAL effectors which benefit xanthomonads by altering host gene transcription, the non-TAL effectors appear to function in suppression of host innate immunity, presumably by interfering with signaling events. The biochemical functions of the majority of non-TAL effectors are unknown, although a few of them are known to have enzymatic activity. AvrBs2 has putative glycerophosphoryldiester phosphodiesterase activity [6] whereas XopQ is predicted to have a inosine-uridine nucleoside N-ribohydrolase activity [7].

The role of individual non-TAL effectors of xanthomonads in suppression of host innate immunity and promotion of virulence has been examined. The non-TAL effectors of *Xanthomonas campestris* pv. *vesicatoria* such as XopN [8], XopJ [9], XopB and XopS [10] have been shown to suppress PAMP induced innate immune responses such as callose deposition in host plants. Suppression of plant defense responses has been also reported by *X. campestris* pv. *vesicatoria* effector XopX when expressed ectopically in *Nicotiana* *benthamiana* [11] and XopD, a SUMO protease, that promotes pathogen growth at later stages of infection in the host plant tomato [12]. In addition, upon ectopic expression, *X* *. oryzae* pv. *oryzae* effectors XopZ [13] and XopR [14] have been reported to suppress PAMP induced callose deposition in *N* *. benthamiana* and *Arabidopsis* respectively. Furthermore, mutations in many of these effector proteins renders the pathogen partially virulence deficient and impaired in symptom development on their respective hosts. Other T3S secreted non-TAL effectors which have been reported to have a role in virulence and symptom development are HpaF [15], AvrXv4 [16] and AvrXccC [17]. In contrast to the large body of work that is available on suppression of PTI, very little information is available about the functions involved in suppression of DAMP triggered innate immunity (DTI) either in xanthomonads or in other plant pathogens.

The plant cell wall is a formidable barrier for plant pathogens. Plant cell wall degradation is a key aspect of microbial plant pathogenesis. Plant pathogenic fungi and bacteria secrete a cocktail of specific hydrolytic enzymes like cellulases, xylanases, polygalacturonases, pectate lyases, pectin esterases etc. to sever different components of the plant cell wall [18]. However, these cell wall degrading enzymes are double edged swords, as the cell wall degradation products that are released by their action serve as marks of infection that are sensed by the plant as DAMPs and results in activation of potent innate immune responses [19,20].

The rice pathogen, *Xanthomonas* *oryzae* pv. *oryzae* , secretes several cell wall degrading enzymes including a lipase/esterase (LipA), cellulase (ClsA), cellobiosidase (CbsA), and xylanase (XynB), using a Type 2 secretion (T2S) system [21–23]. Mutations in genes for each of these individual T2S secreted proteins cause a reduction in *X* *. oryzae* pv. *oryzae* virulence indicating that they are important for the ability of this pathogen to cause disease. Purified preparations of enzymes LipA, ClsA and CbsA have been shown to induce innate immune responses such as callose deposition and programmed cell death in rice tissues [22]. Enzyme activity is required for induction of innate immunity as seen from the observation that a point mutation (S176A) in the active site of the LipA protein results in loss of enzyme activity and ability to induce immune responses [23]. *X* *. oryzae* pv. *oryzae* can suppress cell wall damage induced innate immune responses using a T3S system. The T3S^-^ mutant is itself an inducer of innate immunity and a T2S^-^ T3S^-^ double mutant is compromised in induction of innate immunity suggesting that T2S secreted proteins play an important role in induction of innate immunity during infection [22]. The *X* *. oryzae* pv. *oryzae* T3S secreted effectors (T3Es) that are involved in suppression of cell wall damage induced rice innate immunity have not yet been identified.

In this study, we tested the ability of 16 different T3S secreted non-TAL effectors of *X* *. oryzae* pv. *oryzae* [24] to suppress cell wall damage induced innate immunity in rice. We found that *Agrobacterium* mediated transient transfer of *xopN* gene of *X* *. oryzae* pv. *oryzae* suppressed cell wall damage induced programmed cell death in rice roots and callose deposition in rice leaves mediated by enzyme LipA. However, the ability of a *xopN* ^*-*^ mutant to suppress innate immunity induced by LipA indicated the presence of other functionally redundant T3S effectors. We thus screened 15 other T3S secreted proteins of *X* *. oryzae* pv. *oryzae* and identified XopQ, XopX and XopZ proteins as suppressors of LipA induced innate immunity in rice. For the remaining 12 effectors, namely XopF, XopK, XopP, XopR, XopT, XopU, XopV, XopW, XopY, XopAA, XopAB and AvrBs2, no evidence for suppressor activity was found although it cannot be completely ruled out. This is the first report showing role of bacterial T3Es in suppression of DTI in host-pathogen interactions.

## Results

### XopN protein of *X* *. oryzae* pv. *oryzae* suppresses cell wall damage induced programmed cell death in rice roots

In a previously published study of *Xanthomonas* T3S effectors, mutation of the *xopN* gene of the tomato pathogen *X. campestris* pv. *vesicatoria* was found to affect virulence [7]. It had also been suggested, based on the presence of ARM/HEAT repeats, that the XopN protein might modulate plant signal transduction by interfering with host proteins containing these repeats. Therefore, we decided to first address the ability of *X* *. oryzae* pv. *oryzae* XopN protein to suppress host defence responses induced by cell wall degrading enzyme, LipA.

**Figure 1:**
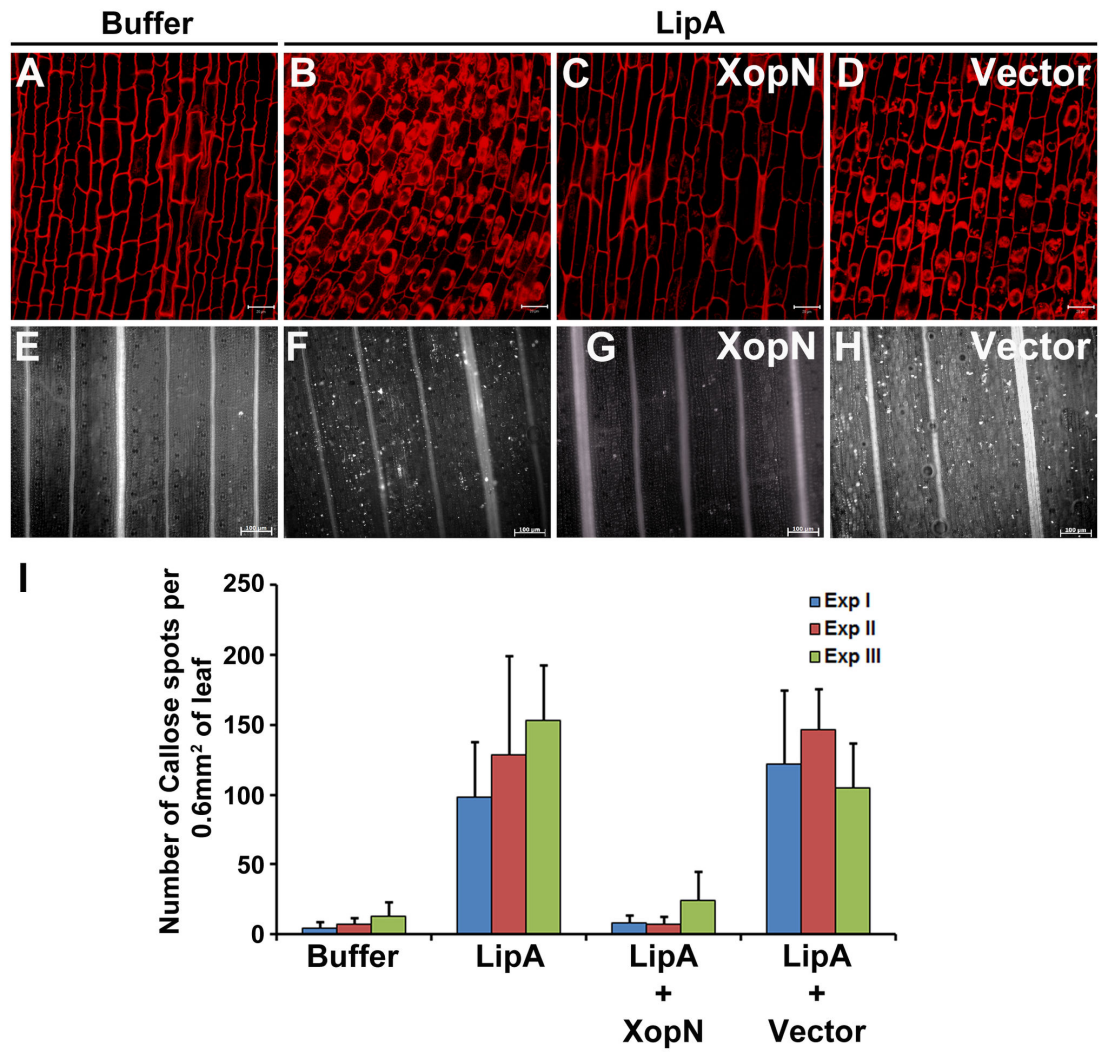
*Agrobacterium* mediated transient transfer of the *xopN* gene of *Xanthomonas* *oryzae* pv. *oryzae* suppresses LipA induced programmed cell death in rice roots and callose deposition in rice leaves. (**A**) to (**D**) Rice roots were treated with one of the following: buffer (**A**); LipA (**B**); pretreatment with EHA105/p*Ub*i-*xopN* followed by treatment with LipA (**C**); pretreatment with EHA105/p*Ub*i followed by treatment with LipA (**D**). Treated roots were subsequently stained with propidium iodide (PI) and sectioned using confocal microscopy. Internalisation of PI is indicative of defense response-associated programmed cell death in rice roots. Scale bar measures 20µm. (**E**) to (**H**) Rice leaves were infiltrated with one of the following: buffer (**E**); LipA (**F**); EHA105/p*Ub*i-*xopN* along with LipA (**G**); EHA105/p*Ub*i along with LipA (**H**). The treated leaves were subsequently stained with aniline blue and visualized under an epifluorescence microscope. White dots in these pictures are indicative of callose deposition. Scale bar measures 100µm. (**I**) Mean and standard deviation were calculated for number of callose deposits observed in an area of 0.60 mm^2^. Data were collected from at least five leaves per treatment in each experiment (three experiments indicated as ExpI, ExpII and ExpIII) and two to three different viewing areas from the infiltrated region of each leaf. Statistically significant differences at *P* < 0.05 (Student’s two-tailed *t* test for independent means) were obtained from leaves infiltrated with EHA105/p*Ub*i-*xopN* along with LipA as compared to leaves treated with LipA alone. These differences were not observed in leaves infiltrated with EHA105/p*Ub*i along with LipA as compared to leaves treated with LipA alone. WT Xoo = wild type *X* *. oryzae* pv. *oryzae* . WT Xoo = wild type *X* *. oryzae* pv. *oryzae* .

Localized programmed cell death (PCD) is an important host defence response against pathogen attack [25]. In a previous study, it was shown that a PCD is induced in rice roots by treatment with *X* *. oryzae* pv. *oryzae* secreted hydrolytic enzymes such as LipA (Lipase/esterase A) [22]. The extent of PCD was assessed by staining with propidium iodide (PI) and visualization under a confocal laser scanning microscope. We cloned the *xopN* gene into the MCS (multiple cloning site) of the binary vector p*Ubi*-C1300 [26] wherein its expression is under the control of the maize ubiquitin promoter. Rice roots treated with LipA (Figure 1B) took up PI and the PI stained material was dispersed within the cell, indicating nuclear fragmentation. Buffer (Figure 1A) treated rice roots excluded PI and showed only cell wall associated fluorescence. Rice roots that were pre-treated with *Agrobacterium* strain EHA105/p*Ubi*-*xopN* followed by treatment with LipA (Figure 1C) excluded PI and exhibited only cell wall associated fluorescence indicating absence of any cell death. In contrast, LipA (Figure 1D) treatment caused extensive cell death in roots which had been pre-treated with *Agrobacterium* strain EHA105/p*Ubi* (empty vector).

RT-PCR analysis was performed to confirm that *xopN* transcripts were expressed in rice roots treated with EHA105/p*Ubi*-*xopN* (Figure S1). A 123 bp product was obtained after RT-PCR with RNA isolated from rice roots treated with EHA105/p*Ubi*-*xopN* but was not observed in roots treated with EHA105/p*Ubi*. Also, as expected, a 158 bp RT-PCR product of the rice *GAPDH* gene was obtained from rice roots that were pre-treated with *Agrobacterium* (either EHA105/p*Ubi*-*xopN* or EHA105/p*Ubi*) (Figure S1A).

### XopN protein of *X* *. oryzae* pv. *oryzae* suppresses LipA induced callose deposition in rice leaves

Callose deposition is a basal defense response to strengthen the cell wall and is characterized by deposition of a β-1, 3-glucan polymer around the site of pathogen entry [27]. Upon aniline blue staining, callose deposits appear as bright spots when viewed under an epifluorescence microscope. Infiltration of LipA results in induction of callose deposition in rice leaves [22]. We assessed whether *Agrobacterium* mediated transient transfer of the *xopN* gene would result in suppression of LipA induced callose deposition. Rice leaves, hand infiltrated with purified LipA (Figure 1F) exhibited substantially higher amounts of callose deposits compared with leaves infiltrated with buffer (Figure 1E) alone. Co-infiltration of LipA (Figure 1G) along with *Agrobacterium* strain EHA105/p*Ubi*-*xopN* resulted in suppression of callose deposition to a level that is observed in buffer (Figure 1E) infiltrated leaves. Rice leaves co-infiltrated with LipA (Figure 1H) and *Agrobacterium* strain EHA105/p*Ubi* (empty vector) exhibited higher amounts of callose deposits that were no different from those observed in leaves infiltrated with LipA (Figure 1F) alone (Figure 1I). These data indicated that transient transfer of the *xopN* gene of *X* *. oryzae* pv. *oryzae* suppresses callose deposition that is induced by treatment with cell wall degrading enzyme (LipA) in rice leaves.

### A *xopN* ^-^ mutant of *X* *. oryzae* pv. *oryzae* retains the ability to suppress rice innate immunity

**Figure 2:**
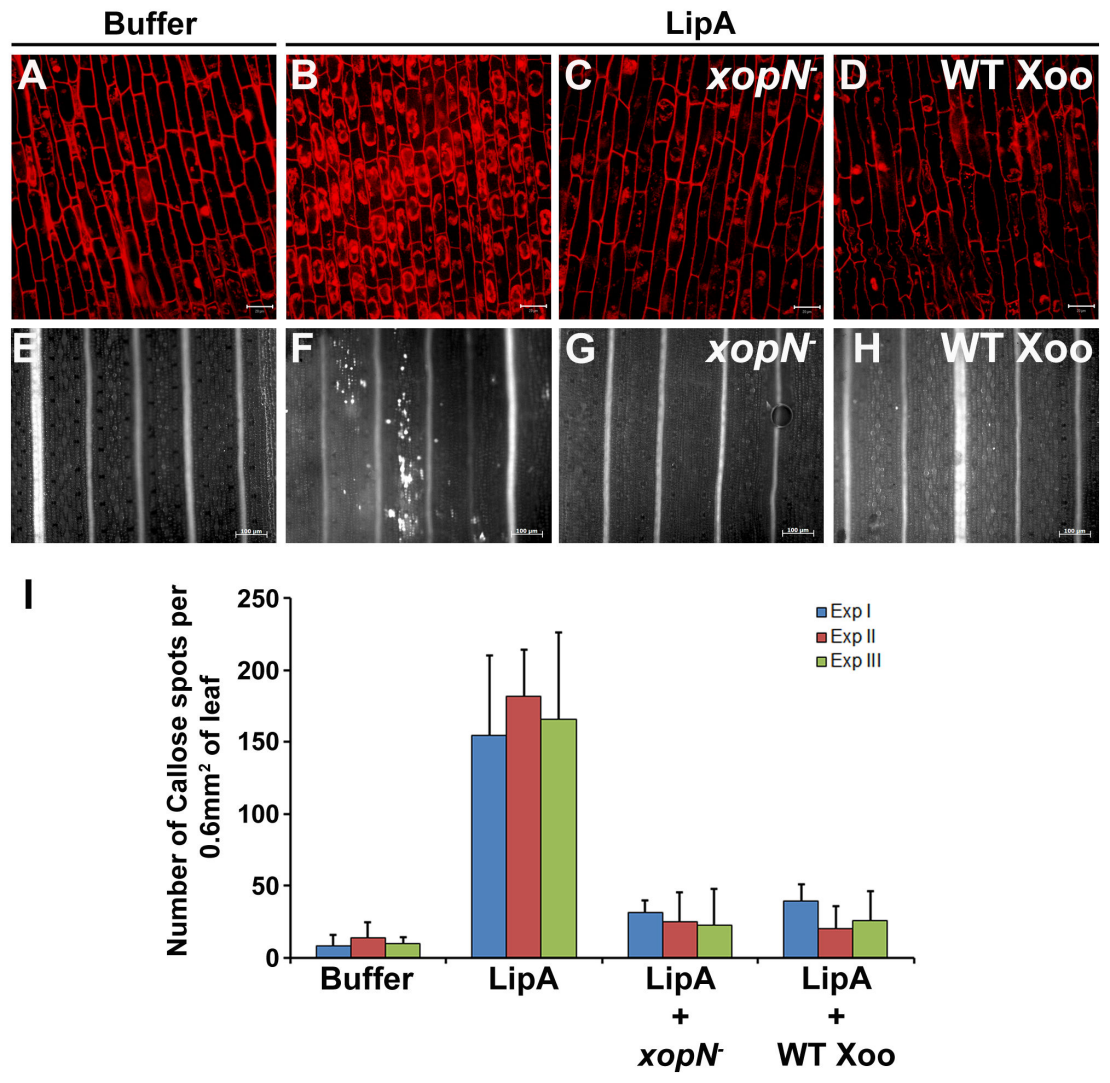
A *xopN* ^*-*^ mutant of *Xanthomonas* *oryzae* pv. *oryzae* is proficient in suppression of LipA induced cell death in rice roots and callose deposition in rice leaves. (**A**) to (**D**) Rice roots were treated with one of the following: buffer (**A**); LipA (**B**); pretreatment with *xopN* ^*-*^ mutant followed by treatment with LipA (**C**); pretreatment with wild type followed by treatment with LipA (**D**). Treated roots were subsequently stained with propidium iodide (PI) and viewed under a confocal microscope. Internalisation of PI is indicative of defense response-associated programmed cell death. Scale bar measures 20µm. (**E**) to (**H**) Rice leaves were infiltrated with one of the following: buffer (**E**); LipA (**F**); *xopN* ^*-*^ mutant along with LipA (**G**); wild type along with LipA (**H**). The infiltrated leaves were stained for callose and visualised under an epifluorescence microscope. Callose deposits appeared as sharp white spots in the viewed field. Scale bar measures 100µm. (**I**) Mean and standard deviation were calculated for number of callose deposits from an area of 0.60 mm^2^. Data were collected from at least five leaves per treatment in each experiment (three experiments indicated as ExpI, ExpII and ExpIII) and two to three different viewing areas from the infiltrated region of each leaf. Statistically significant differences at *P* < 0.05 (Student’s two-tailed *t* test for independent means) were observed in values obtained from leaves infiltrated with *xopN* ^-^ mutant (or wild type) along with LipA as compared to leaves infiltrated with LipA alone. WT Xoo = wild type *X* *. oryzae* pv. *oryzae* .

A *xopN* gene disruption mutant of *X* *. oryzae* pv. *oryzae* (*xopN* ^-^) was isolated by homologous plasmid integration. We next tested the ability of a *X* *. oryzae* pv. *oryzae* *xopN* ^-^ mutant to suppress LipA induced innate immunity. To address this issue, rice roots were pre-treated with either *xopN* ^-^ or wild type followed by treatment with purified LipA. The roots were then stained with PI and viewed under a confocal laser scanning microscope. As expected, roots treated with buffer (Figure 2A) exhibited only cell wall associated fluorescence whereas roots treated with purified LipA (Figure 2B) exhibited extensive cell death. Interestingly, pretreatment with either the *xopN* ^-^ (Figure 2C) or wild type (Figure 2D) strains resulted in suppression of LipA induced cell death. Although some of the cells took up PI, the extent of intracellular dispersion of PI staining material was limited as compared to that observed in roots treated with LipA (Figure 2B) alone. This prompted us to check if the *X* *. oryzae* pv. *oryzae* *xopN* ^*-*^ mutant can also suppress innate immune response associated callose deposition. Co-infiltration of rice leaves with either *xopN* ^-^ (Figure 2G) or wild type (Figure 2H) along with LipA resulted in lesser amounts of callose deposition as compared to infiltration with LipA (Figure 2F) alone. This indicated that a *xopN* ^-^ mutant is proficient in suppression of LipA induced callose deposition in rice leaves (Figure 2I).

### Transient expression of *xopQ*, *xopX* or *xopZ* genes of *X* *. oryzae* pv. *oryzae* suppresses cell wall damage induced PCD in rice roots

**Figure 3:**
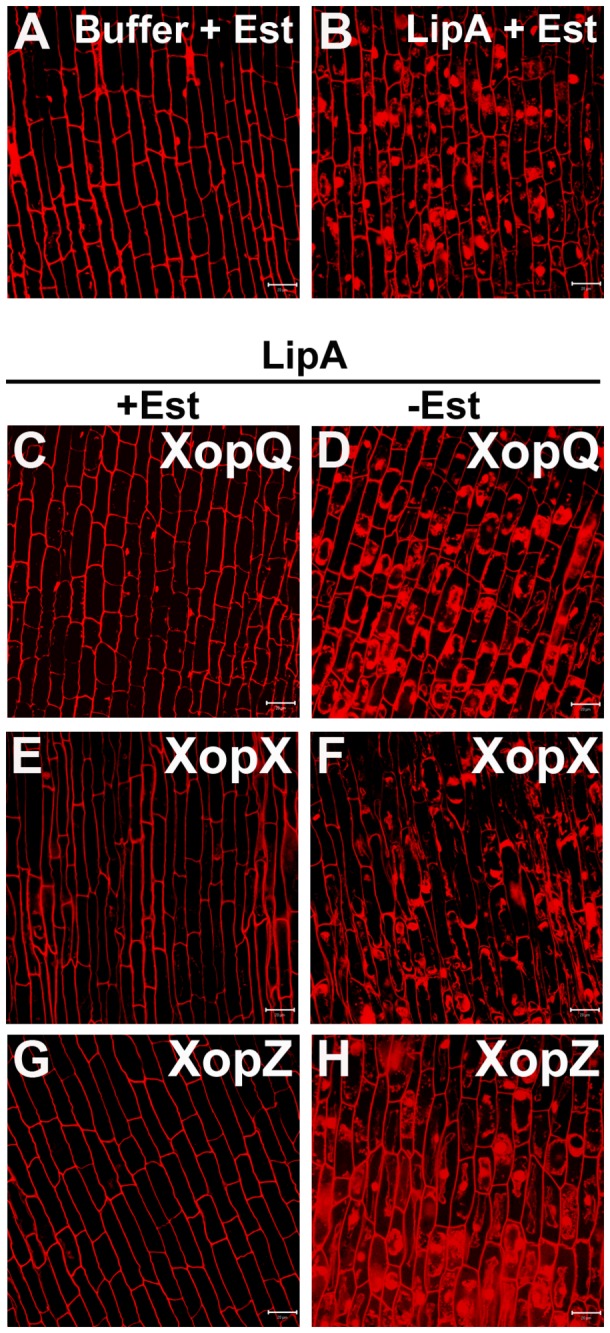
*Agrobacterium* mediated transient transfer of *xopQ*, *xopX* and *xopZ* genes of *Xanthomonas* *oryzae* pv. *oryzae* suppresses LipA induced programmed cell death in rice roots. Rice roots were treated with the following: buffer + Estradiol (Est; **A**); LipA + Est (**B**); pretreatment with EHA105/pMDC7-*xopQ* with Est (**C**); EHA105/pMDC7-*xopX* with Est (**E**) and EHA105/pMDC7-*xopZ* with Est (**G**) followed by treatment with LipA and Est (**C**, **E** and **G**); pretreatment with EHA105/pMDC7-*xopQ* (**D**); EHA105/pMDC7-*xopX* (**F**) and EHA105/pMDC7-*xopZ* (**H**) followed by treatment with LipA (**D**, **F** and **H**). The roots were subsequently stained with propidium iodide (PI) and visualized under a confocal microscope. Dispersed intracellular PI staining is indicative of programmed cell death in rice roots. Scale bar measures 20µm.

The ability of a *X* *. oryzae* pv. *oryzae* *xopN* ^-^ mutant to suppress innate immune responses induced by LipA indicated the presence of other functionally redundant T3S effectors. We therefore assessed the ability of 15 non-TAL T3S effectors of *X* *. oryzae* pv. *oryzae* , from the set of previously identified *X* *. oryzae* pv. *oryzae* T3S effectors [24], to suppress LipA induced PCD in rice roots. The genes for 15 of these non-TAL T3S effectors (*xopF*, *xopK*, *xopP*, *xopQ*, *xopR*, *xopT*, *xopU*, *xopV, xopW*, *xopX, xopY, xopZ, xopAA, xopAB* and *avrBs2*) were cloned into the Agrobacterial T-DNA vector as described in materials and methods. Rice roots were individually pretreated with *Agrobacterium* strain EHA105 carrying the genes for each of these individual T3S effectors cloned into a T-DNA vector. These roots were subsequently treated with LipA enzyme followed by PI staining and sectioning under confocal laser scanning microscope. As expected, rice roots treated with LipA + 17-β-estradiol (Est) (Figure 3B) exhibited dispersed intracellular PI staining indicating extensive cell death whereas buffer (+Est) (Figure 3A) treated roots showed only cell wall associated fluorescence indicating no cell death. The rice roots pretreated with *Agrobacterium* strain EHA105 carrying pMDC7-*xopQ* (Figure 3C), pMDC7-*xopX* (Figure 3E) or pMDC7-*xopZ* (Figure 3G) in the presence of Est followed by LipA treatment exhibited cell wall associated fluorescence with no intracellular PI staining. However, in the absence of Est, roots pretreated with these strains followed by LipA treatment showed dispersed intracellular PI staining indicating extensive cell death (Figure 3D, F and H respectively). This indicates that induction of expression of *xopQ*, *xopX* or *xopZ* genes is sufficient for suppression of LipA induced PCD. Pretreatment with EHA105 carrying Agrobacterial T-DNA vector with any of the following genes *xopF*, *xopK*, *xopP*, *xopR*, *xopT*, *xopU*, *xopV, xopW*, *xopY, xopAA, xopAB* or *avrBs2* did not result in suppression of PCD either in the presence or absence of Est (data not shown). Thus, only XopQ, XopX and XopZ appear to function as suppressors of LipA induced PCD in rice roots. RT-PCR analysis was performed to confirm that *xopQ, xopR, xopX and xopZ* transcripts were expressed in rice roots treated with EHA105/pMDC7-*xopQ/R/X/Z* gene only in the presence of estradiol (Figure S1B). RT-PCR analysis also indicated that the *xopP* gene is expressed in rice roots following transient assays using EHA105/p*Ubi*-*xopP* (Figure S1A). The *xopP* and *xopR* genes were chosen for expression analysis as representatives of T3S effectors which do not suppress cell death in the transient transfer assay. The results indicate that, at least for *xopP* and *xopR* and possibly for the other T3S effectors that do not suppress cell death, the absence of suppression is not due to lack of expression of the gene in rice roots during transient assays.

### Transient expression of *xopQ*, *xopX* or *xopZ* genes of *X* *. oryzae* pv. *oryzae* suppresses cell wall damage induced callose deposition in rice leaves

**Figure 4:**
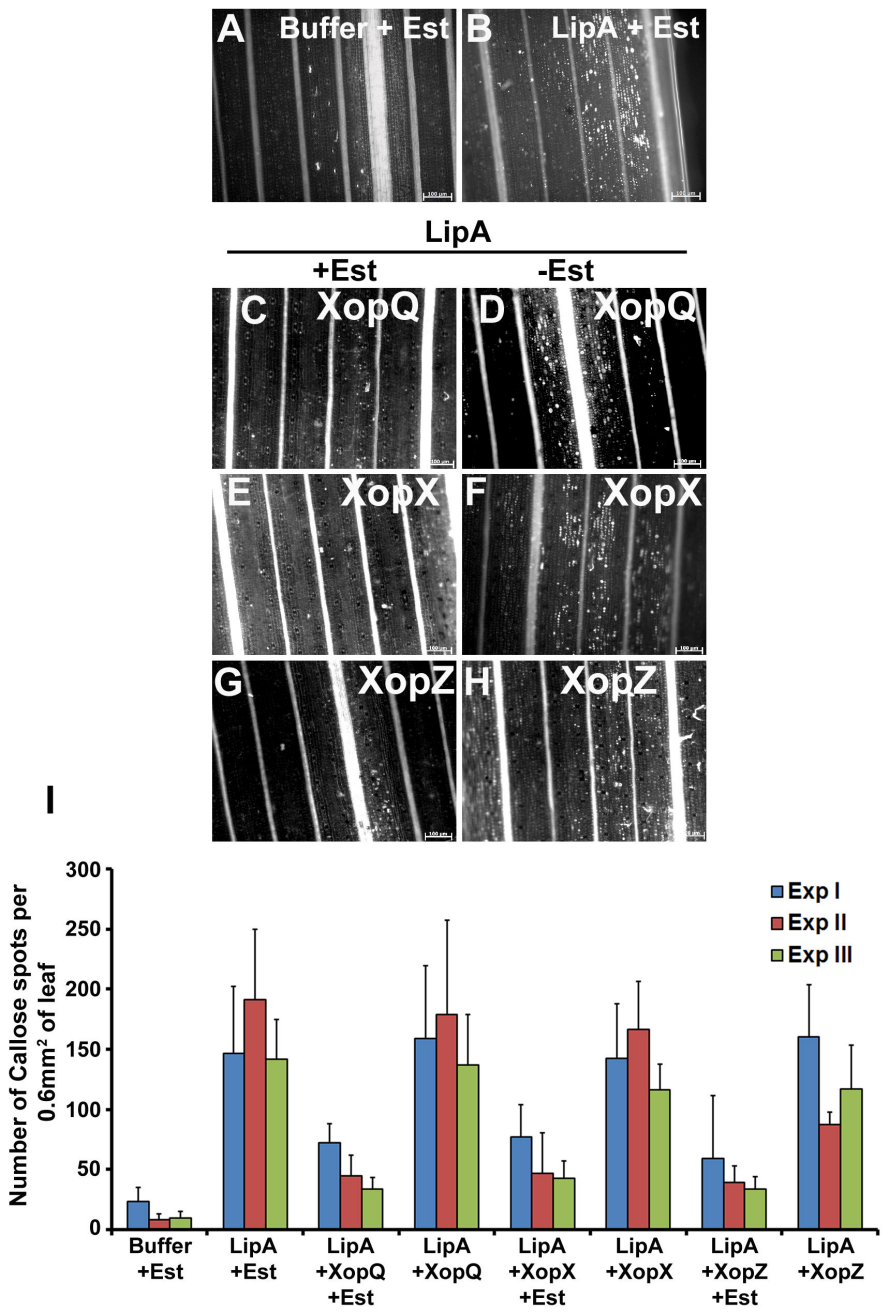
*Agrobacterium* mediated transient transfer of *xopQ*, *xopX* and *xopZ* genes of *Xanthomonas* *oryzae* pv. *oryzae* suppresses LipA induced callose deposition in rice leaves. Rice leaves were infiltrated with one of the following: buffer + Estradiol (Est; **A**); LipA + Est (**B**); EHA105/pMDC7-*xopQ* + LipA with Est (**C**) or without Est (**D**); EHA105/pMDC7-*xopX* + LipA with Est (**E**) or without Est (**F**); EHA105/pMDC7-*xopZ* + LipA with Est (**G**) or without Est (**H**). The leaves were subsequently stained with aniline blue and visualized under an epifluorescence microscope. Callose deposition is seen as white spots in these pictures. Scale bar measures 100µm. (**I**) Mean and standard deviation were calculated for number of callose deposits observed in an area of 0.60 mm^2^. Data were collected from at least five leaves per treatment in each experiment (three experiments indicated as ExpI, ExpII and ExpIII) and two to three different viewing areas from the infiltrated region of each leaf. Statistically significant differences at *P* < 0.05 (Student’s two-tailed *t* test for independent means) were obtained from leaves co-infiltrated with LipA and EHA105 containing pMDC7-*xopQ*, pMDC7-*xopX* or pMDC7-*xopZ* in the presence of Est as compared to the absence of Est.

We then proceeded to assess whether transient transfer of any of these 15 non-TAL effectors would suppress LipA induced callose deposition in rice leaves. Co-infiltration of LipA along with *Agrobacterium* strain EHA105 carrying pMDC7-*xopQ* (Figure 4C), pMDC7-*xopX* (Figure 4E) or pMDC7-*xopZ* (Figure 4G) in the presence of Est resulted in significantly less callose deposition than that observed in LipA and Est (Figure 4B) infiltrated rice leaves. In contrast to this, rice leaves co infiltrated with LipA along with *Agrobacterium* strain EHA105 carrying pMDC7-*xopQ* (Figure 4D), pMDC7-*xopX* (Figure 4F) or pMDC7-*xopZ* (Figure 4H) in the absence of Est exhibited levels of callose deposits that were similar to those observed in leaves infiltrated with LipA and Est (Figure 4B) (Figure 4I). As observed for the suppression of LipA induced PCD in rice roots, co-infiltration of LipA and EHA105/carrying Agrobacterial T-DNA vector with any of the following genes *xopF*, *xopK*, *xopP*, *xopR*, *xopT*, *xopU*, *xopV, xopW*, *xopY, xopAA, xopAB* or *avrBs2* did not result in suppression of callose deposition either in the presence or absence of Est (data not shown). These data indicated that *xopX*, *xopQ* or *xopZ* genes were able to suppress LipA induced callose deposition in rice leaves.

### A *xopN* ^-^ *xopQ* ^-^ *xopX* ^-^ *xopZ* ^-^ quadruple mutant of *X* *. oryzae* pv. *oryzae* induces callose deposition in rice leaves

**Figure 5:**
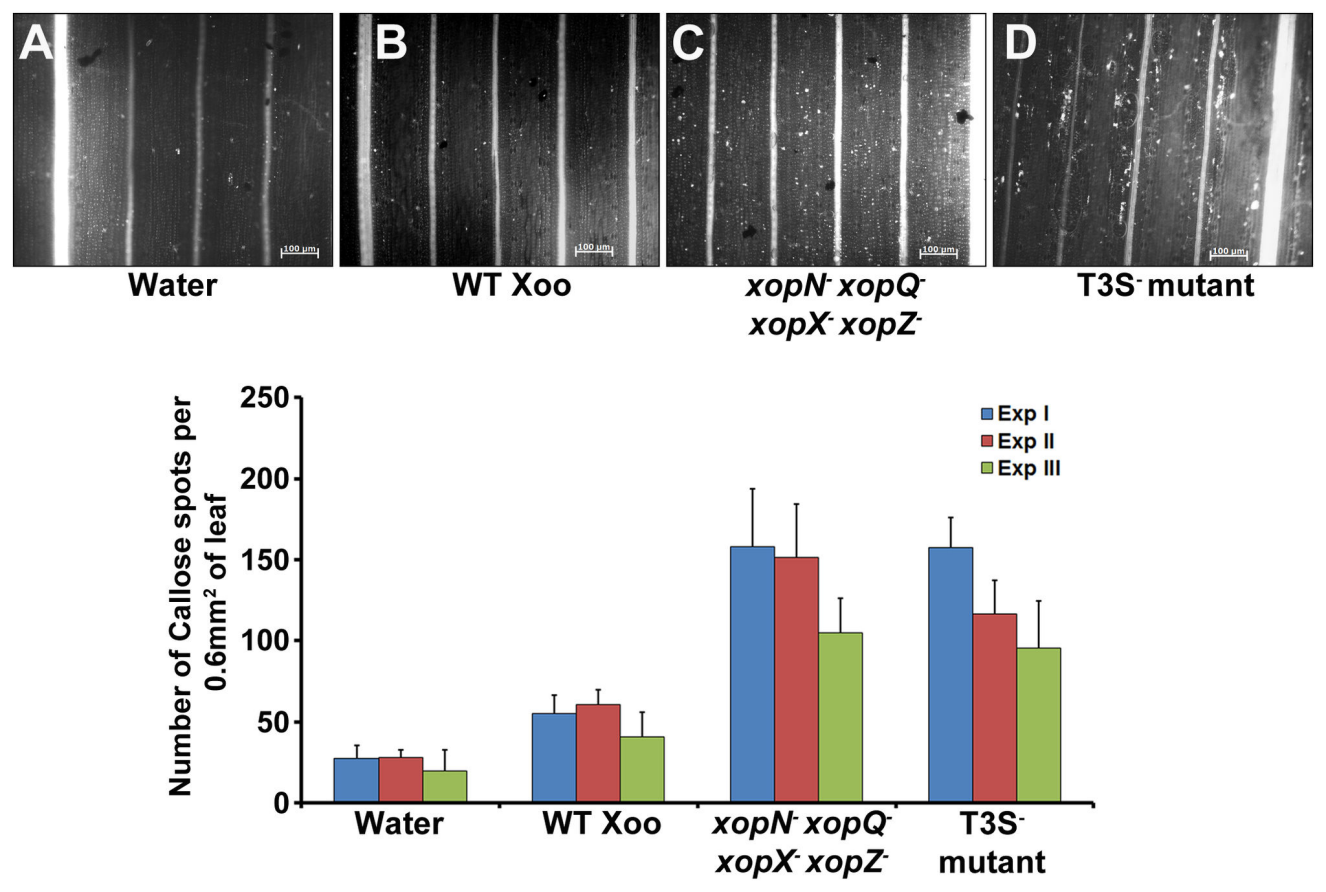
A *xopN* ^*-*^ *xopQ* ^*-*^ *xopX* ^*-*^ *xopZ* ^*-*^ quadruple mutant of *Xanthomonas* *oryzae* pv. *oryzae* induces callose deposition in rice leaves. Rice leaves were infiltrated with one of the following: water (**A**); wild type Xoo (**B**); *xopN* ^*-*^ *xopQ* ^*-*^ *xopX* ^*-*^ *xopZ* ^*-*^ quadruple mutant (**C**) and T3S^-^ mutant (**D**). The treated leaves were subsequently stained with aniline blue and visualized under an epifluorescence microscope. White dots in these pictures are indicative of callose deposition. Scale bar measures 100µm. (**E**) Mean and standard deviation were calculated for number of callose deposits observed in a leaf area of 0.60 mm^2^. Data were collected from at least five leaves per treatment in each experiment (three experiments indicated as ExpI, ExpII and ExpIII) and two to three different viewing areas from the infiltrated region of each leaf. Statistically significant differences at *P* < 0.05 (Student’s two-tailed *t* test for independent means) were obtained from leaves infiltrated with a *xopN* ^*-*^ *xopQ* ^*-*^ *xopX* ^*-*^ *xopZ* ^*-*^ quadruple mutant as compared to leaves treated with either water or wild type. These differences were not observed in leaves infiltrated with *xopN* ^*-*^ *xopQ* ^*-*^ *xopX* ^*-*^ *xopZ* ^*-*^ quadruple mutant as compared to leaves treated with T3S^-^ mutant. WT Xoo = wild type *X* *. oryzae* pv. *oryzae* .

A T3S deficient strain of *X* *. oryzae* pv. *oryzae* has been shown to be an inducer of innate immune responses in rice [22]. This has been attributed to its inability to suppress host immune responses induced by DAMPs and possibly PAMPs. The experiments described above indicate that the T3S effectors XopN, XopQ, XopX and XopZ are involved in suppression of LipA induced innate immunity in rice. This prompted us to construct a *xopN* ^*-*^ *xopQ* ^*-*^ *xopX* ^*-*^ *xopZ* ^*-*^ quadruple mutant of *X* *. oryzae* pv. *oryzae* and determine if it is able to, like a T3S^-^ mutant, induce innate immunity in rice. The ability to induce callose deposition was assessed by infiltrating rice leaves with wild type *X* *. oryzae* pv. *oryzae* , *xopN* ^*-*^ *xopQ* ^*-*^ *xopX* ^*-*^ *xopZ* ^*-*^ quadruple mutant or T3S^-^ mutant. Infiltration of wild type *X* *. oryzae* pv. *oryzae* (Figure 5B) resulted in induction of a basal level of callose deposition that is no different from that observed in water (Figure 5A) infiltrated leaves. Rice leaves infiltrated with *xopN* ^*-*^ *xopQ* ^*-*^ *xopX* ^*-*^ *xopZ* ^*-*^ quadruple mutant (Figure 5C) exhibited higher amounts of callose deposits which were similar in amounts to those observed in leaves infiltrated with the T3S^-^ mutant (Figure 5D). In addition, like the T3S^-^ mutant, the *xopN* ^*-*^ *xopQ* ^*-*^ *xopX* ^*-*^ *xopZ* ^*-*^ quadruple mutant induced PCD in rice roots (Figure S2). These data indicate that a *X* *. oryzae* pv. *oryzae* quadruple mutant that is defective in all four of the *xopN, xopQ, xopX* and *xopZ* genes induces innate immune responses such as callose deposition and PCD to the same extent as a T3S^-^ mutant strain of *X* *. oryzae* pv. *oryzae* *.*

In order to assess the effect of addition of a single effector to the quadruple mutant, we introduced the *xopN* gene into the *xopN* ^*-*^ *xopQ* ^*-*^ *xopX* ^*-*^ *xopZ* ^*-*^ quadruple mutant and assessed the ability of the *xopN* complemented strain to induce callose deposition in rice leaves. Rice leaves infiltrated with either *xopN* ^*-*^ *xopQ* ^*-*^ *xopX* ^*-*^ *xopZ* ^*-*^ **/** *xopN* ^*+*^ or *xopQ* ^*-*^ *xopX* ^*-*^ *xopZ* ^*-*^ triple mutant exhibited similar amounts of callose deposits which were lower than those observed in leaves infiltrated with *xopN* ^*-*^ *xopQ* ^*-*^ *xopX* ^*-*^ *xopZ* ^*-*^ quadruple mutant. Infiltration of a *xopN* ^*-*^ *xopQ* ^*-*^ *xopX* ^*-*^ *xopZ* ^*-*^ **/**pHM1 (vector control) strain resulted in callose amounts similar to those observed in *xopN* ^*-*^ *xopQ* ^*-*^ *xopX* ^*-*^ *xopZ* ^*-*^ infiltrated leaves (Figure S3). These data indicate that the enhanced ability of the quadruple mutant to induce immune responses is due to loss of an intact *xopN* gene and not due to a strange recombination event that might have occurred during creation of the *xopN* mutation in the triple mutant background during generation of the quadruple mutant. Furthermore, the ability to induce callose deposition was assessed for *X* *. oryzae* pv. *oryzae* *xopN* ^*-*^, *xopQ* ^*-*^, *xopX* ^*-*^ or *xopZ* ^*-*^ single mutant strains. The number of callose deposits in rice leaves infiltrated with any single *X* *. oryzae* pv. *oryzae* effector mutant was comparable to those observed in wild type infiltrated leaves (Figure S4). Similarly, the number of callose deposits in rice leaves infiltrated with either one of two double mutants (*xopN* ^*-*^ *xopX* ^*-*^ and *xopQ* ^*-*^ *xopX* ^*-*^) or the triple mutant (*xopQ* ^*-*^ *xopX* ^*-*^ *xopZ* ^*-*^) were comparable to those observed in wild type infiltrated leaves (data not shown). These data demonstrate that multiple T3S effectors are required for full suppression of rice immune responses.

### Mutations in *xopN, xopQ, xopX* and *xopZ* genes affect virulence of *X* *. oryzae* pv. *oryzae* on rice

**Figure 6:**
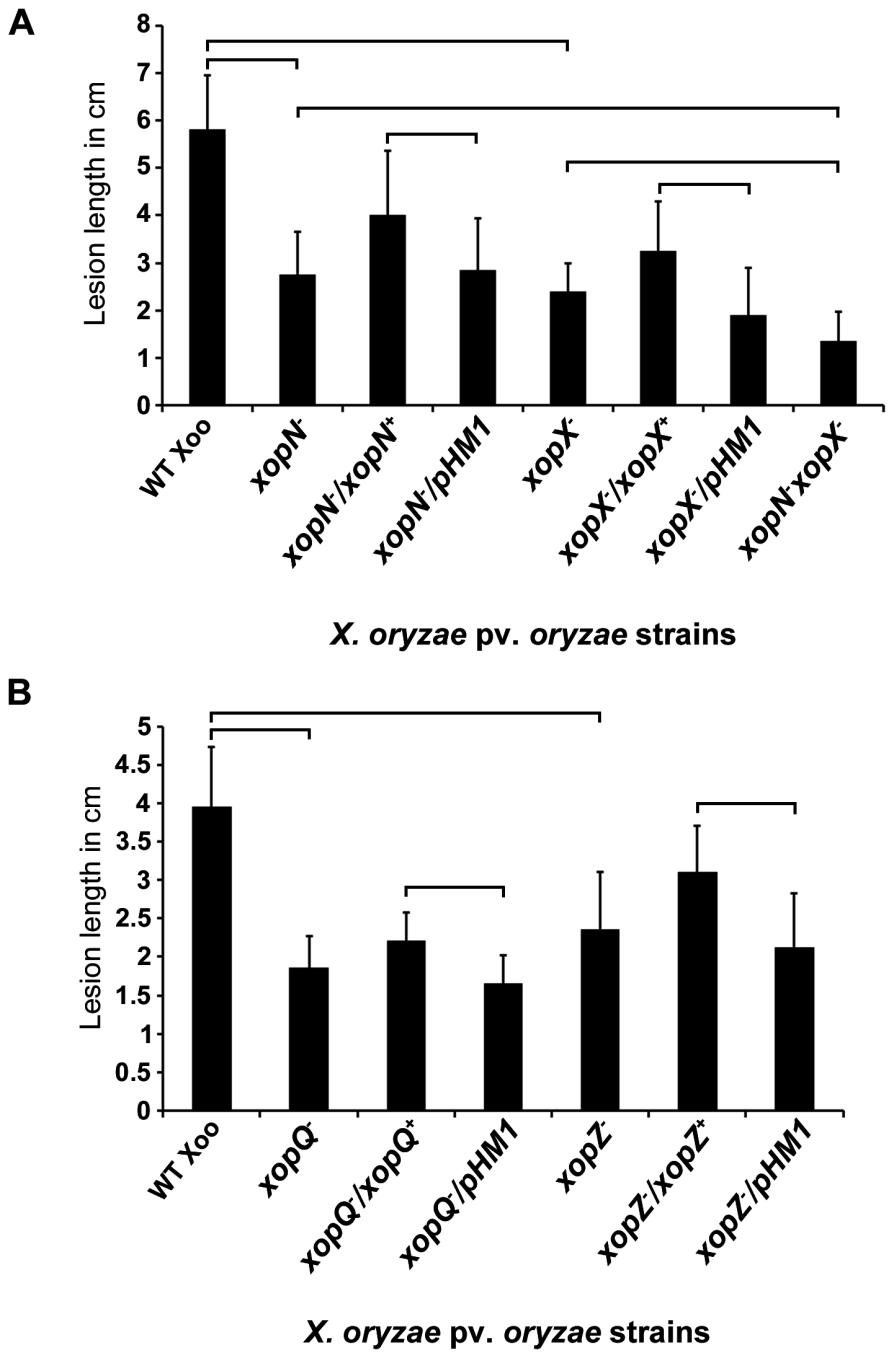
Virulence deficiency associated with *xopN* ^*-*^, *xopQ* ^*-*^, *xopX* ^*-*^ or *xopZ* ^*-*^ single mutants of *Xanthomonas* *oryzae* pv. *oryzae* . Leaves of susceptible rice variety Taichung-Native 1 (TN-1) were clip inoculated with the following *X* *. oryzae* pv. *oryzae* strains: (**A**) wild type, *xopN* ^*-*^ mutant, *xopN-*/*xopN* ^*+*^ (complemented strain), *xopN* ^*-*^/pHM1 (vector control), *xopX* ^*-*^ mutant, *xopX-*/*xopX* ^*+*^ (complemented strain), *xopX* ^*-*^/pHM1 (vector control) or *xopN* ^*-*^ *xopX* ^*-*^ double mutant strains; (**B**) wild type, *xopQ* ^*-*^ mutant, *xopQ-*/*xopQ* ^*+*^ (complemented strain), *xopQ* ^*-*^/pHM1 (vector control), *xopZ* ^*-*^ mutant, *xopZ-*/*xopZ* ^*+*^ (complemented strain) or *xopZ* ^*-*^/pHM1 (vector control). Lesion lengths were measured 7 days post inoculation. Error bars indicate the standard deviation of readings from at least 10 inoculated leaves. Similar results were obtained in independent experiments. A Student’s two-tailed *t* test for independent means was performed for the following values: wild type with each of the single mutants and the double mutant with each of the single mutants with correction for multiple comparisons, mutant with empty vector and complemented strains for each single mutant. The brackets on the graphs indicate the comparisons that were made. All compared values are significantly different at *P* < 0.05 level.

A *xopX* ^*-*^ mutant of *X* *. oryzae* pv. *oryzae* was isolated by creating an inframe deletion in the *xopX* gene of the wild type strain described in materials and methods. A *xopN* ^*-*^ *xopX* ^*-*^ double mutant of *X* *. oryzae* pv. *oryzae* was created by homologous plasmid integration of pK18*mob-xopN* in the *xopX* ^*-*^ background. The virulence of *X* *. oryzae* pv. *oryzae* strains *xopN* ^*-*^ mutant, *xopN* ^*-*^/*xopN* ^*+*^; which carries *xopN* for complementation, *xopN* ^*-*^/pHM1; vector control, *xopX* ^*-*^ mutant, *xopX* ^*-*^/*xopX* ^*+*^; which carries *xopX* for complementation, *xopX* ^*-*^/pHM1; vector control and *xopN* ^*-*^ *xopX* ^-^ double mutant was assessed by clip inoculation of rice leaves. Lesion lengths were measured 7 days after inoculation (DAI). At 7 DAI, the *xopN* ^*-*^ mutant and *xopX* ^*-*^ mutant strains showed lesions significantly reduced in length as compared to lesions produced by wild type indicating a partial virulence deficiency. The partial virulence deficiency exhibited by *xopN* ^*-*^ mutant and *xopX* ^*-*^ mutants was complemented by the respective genes in trans as the lesions observed in rice leaves clip inoculated with strains *xopN* ^*-*^/*xopN* ^*+*^ and *xopX* ^*-*^/*xopX* ^*+*^ were significantly longer than that seen in rice leaves inoculated with the *xopN* ^*-*^ mutant and *xopX* ^*-*^ mutant strains. The lesion lengths caused by the *xopN* ^*-*^ *xopX* ^-^ double mutant strain were significantly reduced compared to the lesions produced by either *xopN* ^*-*^ mutant or *xopX* ^*-*^ mutant strains (Figure 6A). This demonstrates that mutations in *xopN* and *xopX* genes caused a partial virulence deficiency that can be complemented by the expression of respective genes in trans and a *xopN* ^*-*^ *xopX* ^-^ double mutant exhibited much more pronounced virulence deficiency than either of the single mutants.

The virulence proficiency of strains with single mutations in the *xopQ* and *xopZ* genes was also assessed by inoculation of rice leaves. The results indicate that mutations in either *xopQ* or *xopZ* cause a partial virulence deficiency on rice (Figure 6B). Introduction of complementing clones carrying *xopQ* or *xopZ* genes into their respective mutants leads to enhanced virulence on rice.

## Discussion

DAMPs released following plant cell wall degradation during pathogen attack serve as a mark of infection and induce innate immune responses in the host. These DAMP induced host defense responses are suppressed by the pathogen during infection. Jha et al. [22] showed the involvement of the type 3 secretion system (T3S) of *X* *. oryzae* pv.*oryzae* in suppression of cell wall damage induced innate immune responses in rice. We began our search for T3S effectors (T3Es) of *X* *. oryzae* pv.*oryzae* that can suppress cell wall damage induced innate immune responses of rice by testing each of the 16 T3Es of *X* *. oryzae* pv.*oryzae* that were reported by Furutani and co-workers [24] to be translocated into plant cells. In this study, we have demonstrated that *Agrobacterium* mediated transient expression of any one of the *X* *. oryzae* pv. *oryzae* genes, *xopN, xopQ, xopX and xopZ*, results in suppression of rice innate immune responses such as callose deposition and PCD that are induced by treatment with the cell wall degrading enzyme, LipA. For 12 other genes (namely *xopF, xopK, xopP, xopR, xopT, xopU, xopV, xopW, xopY, xopAA, xopAB* and *avrBs2*-like protein) no evidence of suppressor activity was found under these assay conditions. It is possible that these proteins might function in suppression of innate immune responses induced by either PAMPs [28] or other kinds of DAMPs such as the AtPEP1 peptide [29]. At least for XopR this appears to be true as Akimoto-Tomiyama et al. [14] have demonstrated that XopR is a suppressor of flagellin induced innate immunity in *Arabidopsis* . The possibility that some of these twelve genes might encode functions that act together (for example as a protein complex) in host cells to suppress innate immunity cannot be ruled out as our experimental methodology would not permit us to identify proteins that act in such a fashion.

Jha and co-workers have demonstrated that a T3S^-^ mutant of *X* *. oryzae* pv. *oryzae* functions as an inducer of rice innate immunity [22]. This appears to occur because this mutant is unable to suppress immune responses induced by plant cell wall degrading enzymes that it secretes and also because it produces some other DAMPs or PAMPs. We demonstrate in this paper that a *xopN* ^*-*^ *xopQ* ^*-*^ *xopX* ^*-*^ *xopZ* ^*-*^ quadruple mutant of *X* *. oryzae* pv.*oryzae* induces callose deposition and PCD to the same extent as a T3S^-^ mutant. This indicates that the *xopN*, *xopQ*, *xopX* and *xopZ* gene products play a major role in the suppression of host innate immunity during infection. *X* *. oryzae* pv. *oryzae* strains having mutations in any one of the genes *xopN, xopQ, xopX or xopZ* retain the ability to suppress LipA induced innate immunity in rice. This indicates a functional redundancy amongst these four gene products with regard to their ability to suppress LipA induced immune responses.

As the suppression of host defence responses is very crucial for the pathogen to cause disease, the T3Es that are suppressors of host innate immunity should have a role in bacterial virulence on plants. We show that single mutations in the *xopN*, *xopQ*, *xopX* or *xopZ* genes cause partial virulence deficiency of *X* *. oryzae* pv.*oryzae* on rice. Song and Yang [13] have earlier shown that *xopZ* deficiency in *X* *. oryzae* pv.*oryzae* strain PXO99^A^ results in partial loss of virulence on rice. Taken together, these results indicate that the *xopN*, *xopQ, xopX* and *xopZ* genes are required for full virulence on rice. A *xopN* ^*-*^ *xopX* ^*-*^ double mutant exhibits an even greater virulence deficiency than either one of the single mutants. This indicates that these effectors may contribute independently in promoting virulence on rice. The virulence deficiency associated with each of the single mutations is in contrast to our observation that the single mutants are proficient at suppression of LipA induced innate immunity. This suggests that each of the four effector genes has a role in suppressing immune responses that are independent of those induced by LipA or cell wall damage. The possibility that each of these four effectors has some other role in promoting virulence of *X* *. oryzae* pv.*oryzae*, other than in suppression of innate immunity, cannot also be ruled out.

Why might *X* *. oryzae* pv.*oryzae* need multiple T3Es to suppress cell wall damage induced innate immunity? The presence of a few functionally redundant proteins can be advantageous to the pathogen as it may ensure suppression of LipA/cell wall damage induced innate immunity even if any one of the effector functions is for some reason compromised. Also, multiple effectors may ensure foolproof suppression of host innate immunity by targeting the host signal transduction pathways at multiple steps. Our results indicate that XopN protein of *X* *. oryzae* pv. *oryzae* suppresses innate immune responses induced by treatment with LipA. We have also shown that XopN suppresses innate immune responses induced by CbsA (cellosiosidase), another cell wall degrading enzyme secreted by *X* *. oryzae* pv. *oryzae* (DIPANWITA SINHA and RAMESH SONTI, unpublished results). This suggests that signaling pathways for DAMPs released by each of these cell wall degrading enzymes might be converging in the cell. Kim and co-workers showed that XopN protein of *X. campestris* pv. *vesicatoria* suppresses PTI in tomato [8]. The fact that XopN suppresses PTI and DTI suggests the possibility that certain plant functions with which XopN can interact are common to signalling pathways induced by PAMPs and DAMPs.

Specific biochemical activities have not yet been demonstrated for XopN, XopX, XopZ and XopQ proteins. However, XopQ is predicted to have nucleoside hydolase (IU-NH) activity [7]. It has been speculated that the XopQ effector might be functioning to perturb signaling cascades that lead to defence responses in the host by scavenging or sequestering a nucleoside that may act as a messenger in signaling/metabolic networks [7]. The XopN protein of *X. campestris* pv.*vesicatoria* shows structural homology to proteins containing tandemly repeated α-helices known as ARM/HEAT repeats [7] and found to interact with a 14-3-3 adapter protein of tomato [8,30]. The ARM/HEAT repeats are typically found in eukaryotic proteins that function in diverse cellular signaling networks such as *Arabidopsis* homologs of nuclear transport protein importin-β [31] and AtCAND1 which functions in regulation of auxin signaling [32]. Furthermore, it has been shown that different isoforms of 14-3-3s interact with bacterial effector proteins in various plant-pathogen interactions to modulate plant innate immunity [30,33,34]. It is possible that the XopN protein of *X* *. oryzae* pv.*oryzae* also interacts with rice 14-3-3s during suppression of innate immunity. XopX is a methionine rich protein whereas the XopZ protein is a conserved hypothetical protein without any known motifs. It is possible that XopX and XopZ proteins do not have specific biochemical activities but exert their effects by disrupting signalling pathways through interaction with specific host proteins.

The non-TAL effectors of xanthomonads have been further categorized into core effectors and non-core/variable effectors [35]. XopR, XopK, XopL, XopN, XopP, XopQ, XopX, XopZ and AvrBs2 have been categorized as core effectors as they are found to be conserved in all *Xanthomonas* spp. (whose genomes have been sequenced) with the exception of *X* *. albilineans* str. GPEPC73 which has none of these effectors [36] and *X. campestris* pv. *armoraciae* which has only XopR and XopP [35]. The core effectors might be important for virulence and pathogen fitness in a wide range of host plants. Variable effectors are not uniformly distributed across xanthomonads suggesting their possible role in specific host-pathogen interactions. The observation that XopN, XopQ, XopX and XopZ effectors are involved in suppression of cell wall damage induced innate immunity and the fact that they are found in a wide variety of xanthomonads points to the possibility that suppression of cell wall damage induced innate immunity is a key prerequisite for successful infection in a large number of plants.

Ectopic expression of the *xopZ* gene of *X* *. oryzae* pv. *oryzae* PXO99^A^ strain suppressed defense-related callose deposition induced by a T3S^-^ mutant of *X* *. oryzae* pv. *oryzae* in a non-host plant *N* *. benthamiana* [13]. *X* *. oryzae* pv. *oryzae* PXO99^A^ strain has two identical paralogs of the *xopZ* gene [37] because of a 212 kb duplication in the PXO99^A^ genome that includes the *xopZ* gene. A double mutant in the *xopZ*1 and *xopZ*2 genes of *X* *. oryzae* pv. *oryzae* , and not either of the single mutants, resulted in reduced lesion formation and growth in rice. Introduction of either one of these two genes complemented the mutant phenotype and restored full virulence to the double mutant. In our study, we have shown that transient expression of *X* *. oryzae* pv. *oryzae* *xopZ* gene in rice results in suppression of cell wall damage induced innate immunity. Our observation that a single mutation in the *xopZ* gene is sufficient to affect virulence indicates that the wild type *X* *. oryzae* pv. *oryzae* (the strain from India used in our study) carries a single copy of *xopZ*. In this respect, the wild type strain used in this study is similar to *X* *. oryzae* pv.*oryzae* strain KACC 100331 from Korea [38] and *X* *. oryzae* pv.*oryzae* strain MAFF 311018 from Japan [39] which have single copies of XopZ. Neither Song and Yang [13] nor Zhao et al. [40] observed a reduction in symptoms for *xopN*, *xopQ* or *xopX* mutants of *X* *. oryzae* pv.*oryzae*. We suggest that some other, as yet unidentified function, present in the genetic backgrounds of the wild type strains used in those studies is redundant with these three effectors and that this function is not present in the genetic background of the wild type strain used in our study.

In summary, we have found that four proteins of *X* *. oryzae* pv. *oryzae* namely XopN, XopQ, XopX and XopZ are suppressors of cell wall damage induced innate immunity in rice. This is the first report showing suppression of cell wall damage induced innate immunity by bacterial T3Es in any plant-pathogen interaction. The mechanisms by which these proteins interfere with the elaboration of cell wall damage induced rice innate immunity remains to be determined.

## Materials and Methods

### Bacterial strains, plasmids and growth conditions

The *E. coli* and *X* *. oryzae* pv. *oryzae* strains used in this study are listed in Table 1. The Agrobacterial strains used in this study are listed in Table 2. The plasmids used in this study are listed in Tables 3, 4 and 5. Oligonucleotide primers used in this study are listed in Tables S1 and S2. *X* *. oryzae* pv. *oryzae* strains were grown at 28°C in peptone sucrose (PS) medium, and *E. coli* strains were grown at 37°C in Luria Bertani (LB) medium as described previously [41]. *Agrobacterium tumefaciens* strains were grown in tryptone yeast extract mannitol (TYM) medium with appropriate antibiotics. The concentrations of antibiotics used in this study were as follows: rifampicin (Rf), 50 µg/ml; ampicillin (Ap), 100 µg/ml; spectinomycin (Sp), 50 µg/ml; kanamycin (Km) 50 µg/ml for *E. coli* and 15 µg/ml for *X* *. oryzae* pv. *oryzae* .

**Table 1:** Bacterial strains used in this study.

**Strains**	**Relevant characteristics^a^**	**Reference/Source**
***E. coli* DH5α**		
DH5α	λ^–^ f80d*lacZ*DM15 D(*lacZYA*-*argF*)*U169 recA1 endA hsdR17* (r_K_^–^ m_K_^–^) *supE44 thi-1 gyrA relA1*	Invitrogen
S17-1	RP4-2 Tc::Mu-Km::Tn*7 pro hsdR recA* Tra^+^ used as a mobilizing strain	[52]
** *X* *. oryzae* pv. *oryzae* **		
BXO1	wild type; Indian isolate	Laboratory*
Wild type	*rif*-2; derivative of BXO1	Laboratory*
*xopN^-^*	*xopN*::pK18*mob rif*-2; Km^r^; XopN^-^, derivative of wild type	This work
*xopN^-^/xopN^+^*	*xopN*::pK18*mob rif*-2/pDS2; *xopN* ^+^, Km^r^, Sp^r^; derivative of *xopN* ^*-*^	This work
*xopN* ^*-*^ */*pHM1	*xopN*::pK18*mob rif*-2/pHM1; Km^r^, Sp^r^; derivative of *xopN* ^*-*^	This work
*xopX^-^*	∆*xopX* (243 aa-in-frame deletion) *rif*-2; XopX^-^, derivative of wild type	This work
*xopX^-^/xopX^+^*	∆*xopX rif*-2/pDS4; *xopX* ^+^, Km^r^, Sp^r^; derivative of *xopX* ^*-*^	This work
*xopX* ^*-*^ */*pHM1	∆*xopX rif*-2/pHM1; Km^r^, Sp^r^; derivative of *xopX* ^*-*^	This work
*xopQ^-^*	∆*xopQ* (171 aa-in-frame deletion) *rif*-2; XopQ^-^, derivative of wild type	This work
*xopQ^-^/xopQ^+^*	∆*xopQ rif*-2/pDS6 XopQ^+^, derivative of *xopQ* ^*-*^	This work
*xopQ* ^*-*^ */*pHM1	∆*xopQ rif*-2/pHM1; XopQ^-^, derivative of *xopQ* ^*-*^	This work
*xopZ^-^*	∆*xopZ* (431 aa-in-frame deletion) *rif*-2; XopZ^-^, derivative of wild type	This work
*xopZ* ^*-*^/*xopZ* ^*+*^	∆*xopZ rif*-2/pDS8; XopZ^+^, derivative of *xopZ* ^*-*^	This work
*xopZ* ^*-*^/pHM1	∆*xopZ rif*-2/pHM1; XopZ^-^, derivative of *xopZ* ^*-*^	This work
*xopN^-^ xopX^-^*	*xopN*::pK18*mob rif*-2; Km^r^; XopN^-^ XopX^-^; derivative of *xopX* ^*-*^	This work
*xopQ^-^ xopX^-^*	∆*xopQ rif*-2; XopQ^-^ XopX^-^, derivative of *xopX* ^*-*^	This work
*xopQ^-^ xopX^-^ xopZ^-^*	∆*xopZ rif*-2; XopQ^-^XopX^-^ XopZ^-^, derivative of *xopQ* ^*-*^ * xopX* ^*-*^	This work
*xopN^-^ xopQ^-^ xopX^-^ xopZ^-^*	*xopN*::pK18*mob rif*-2; Km^r^; XopN^-^ XopQ^-^XopX^-^ XopZ^-^derivative of *xopQ* ^*-*^ * xopX* ^*-*^ * xopZ* ^*-*^	This work
*xopN^-^ xopQ^-^ xopX^-^ xopZ^-^/xopN^+^*	*xopN* ^*-*^ * xopQ* ^*-*^ * xopX* ^*-*^ * xopZ* ^*-*^ * rif*-2/pDS2; XopN^+^, Km^r^, Sp^r^; derivative of *xopN* ^*-*^ * xopQ* ^*-*^ *xopX* ^*-*^ * xopZ* ^*-*^	This work
*xopN* ^*-*^ *xopQ* ^*-*^ * xopX* ^*-*^ * xopZ* ^*-*^ */*pHM1	*xopN* ^*-*^ * xopQ* ^*-*^ * xopX* ^*-*^ * xopZ* ^*-*^ * rif*-2/pHM1; Km^r^, Sp^r^; derivative of *xopN* ^*-*^ * xopQ* ^*-*^ * xopX* ^*-*^ * xopZ* ^*-*^	This work
T3S^-^	*hrpB6*: :*bla rif*-2; T3S^-^ Hrp^-^, Ap^r^ derivative of wild type	[22]

^a^ The *rif*-2 mutation confers resistance to rifampicin; Ap^r^, Km^r^ and Sp^r^ indicate resistance to ampicillin, kanamycin and spectinomycin respectively. * Laboratory collection

**Table 2:** Bacterial strains used in this study.

***A. tumefaciens* Strains**	**Relevant characteristics^a^**	**Reference/Source**
EHA101	C58, pTiBo542; T-region::aph, Km^r^; A281 derivative harbouring pEHA10, T-DNA replaced with nptII, super-virulent	[53]
EHA105	Km^s^, derivative of EHA101	[54]
EHA105/ p*Ubi*-*xopN*	EHA105 carrying plasmid p*Ubi*-*xopN* ; Km^r^	This work
EHA105/ p*Ubi*-*xopP*	EHA105 carrying plasmid p*Ubi*-*xopP* ; Km^r^	This work
EHA105/ p*Ubi*-*xopW*	EHA105 carrying plasmid p*Ubi*-*xopW* ; Km^r^	This work
EHA105/ p*Ubi*-*xopY*	EHA105 carrying plasmid p*Ubi*-*xopY* ; Km^r^	This work
EHA105/ p*Ubi*-*xopT*	EHA105 carrying plasmid p*Ubi*-*xopT* ; Km^r^	This work
EHA105/ p*Ubi*-*xopAA*	EHA105 carrying plasmid p*Ubi*-*xopAA* ; Km^r^	This work
EHA105/ p*Ubi*-*xopAB*	EHA105 carrying plasmid p*Ubi*-*xopAB* ; Km^r^	This work
EHA105/ p*Ubi*	EHA105 carrying plasmid p*Ubi* (vector control); Km^r^	This work
EHA105/ pMDC7-*xopR*	EHA105 carrying plasmid pMDC7-*xopR*; Sp^r^	This work
EHA105/ pMDC7-*xopV*	EHA105 carrying plasmid pMDC7-*xopV*; Sp^r^	This work
EHA105/ pMDC7-*xopK*	EHA105 carrying plasmid pMDC7-*xopK*; Sp^r^	This work
EHA105/ pMDC7-*xopF*	EHA105 carrying plasmid pMDC7-*xopF*; Sp^r^	This work
EHA105/ pMDC7-*xopQ*	EHA105 carrying plasmid pMDC7-*xopQ*; Sp^r^	This work
EHA105/ pMDC7-*xopU*	EHA105 carrying plasmid pMDC7-*xopU*; Sp^r^	This work
EHA105/ pMDC7-*xopX*	EHA105 carrying plasmid pMDC7-*xopX*; Sp^r^	This work
EHA105/ pMDC7-*xopZ*	EHA105 carrying plasmid pMDC7-*xopZ*; Sp^r^	This work
EHA105/ pMDC7-*avrBs2*	EHA105 carrying plasmid pMDC7-*avrBs2*; Sp^r^	This work

^a^ Km^s^ indicates sensitivity to kanamycin. Km^r^ and Sp^r^ indicate resistance to kanamycin and spectinomycin

respectively.

**Table 3:** Plasmids used in this study.

**Plasmids**	**Relevant characteristics^a^**	**Reference/Source**
pK18*mob*	pUC18 derivative; Mob^+^ Tra^-^ Km^r^; does not replicate in Xoo	[45]
pK18*mobSacB*	derivative of pK18*mob*, sucrose sensitive	[45]
pHM1	Broad host range cosmid vector (~13.3 kb); Sp^r^	[48]
P*Ubi*	p*Ubi*-C1300 binary cloning vector; Km^r^	[26]
pMDC7	17-β-estradiol inducible binary cloning vector; Sp^r^	[51]
pENTR/D/TOPO	cloning vector; Km^r^	Invitrogen
pDS1	pK18*mob* with 497-bp internal fragment of *xopN* gene of wild type strain of Xoo cloned into *Sma*I site of pk18*mob*	This work
pDS2	pHM1 with 2225-bp fragment containing full length *xopN* gene from genomic DNA of wild type strain of Xoo cloned into *Hind*III and *Sac*I sites of pHM1; Sp^r^	This work
pDS3	pK18*mobSacB* with 1473-bp fragment of *xopX* gene of wild type strain of Xoo with an inframe internal deletion of 729-bp (243aa) and insertion of 12 bp *Xba*I site cloned into *Hin*dIII site of pk18*mobSacB*	This work
pDS4	pHM1 with 2190-bp fragment containing full length *xopX* gene from genomic DNA of wild type strain of Xoo cloned into *Hind*III and *Sac*I sites of pHM1; Sp^r^	This work
pDS5	pK18*mobSacB* with 882-bp fragment of *xopQ* gene of wild type strain of Xoo with an inframe internal deletion of 513-bp (171aa) and insertion of 12 bp *Xba*I site cloned into *Hin*dIII site of pk18*mobSacB*; Km^r^	This work
pDS6	pHM1 with 1422-bp fragment containing full length *xopQ* gene from genomic DNA of wild type strain of Xoo cloned into *Hind*III and *Sac*I sites of pHM1; Sp^r^	This work
pDS7	pK18*mobSacB* with 2570-bp fragment of *xopZ* gene of wild type strain of Xoo with an inframe internal deletion of 1293-bp (431aa) and insertion of 12 bp *Xba*I site cloned into *Hin*dIII site of pk18*mobSacB*	This work
pDS8	pHM1 with 3894-bp fragment containing full length *xopZ* gene from genomic DNA of wild type strain of Xoo cloned into *Hind*III and *Sac*I sites of pHM1; Sp^r^	This work
p*Ubi*-*xopN*	p*Ubi*-C1300 with 2193-bp fragment containing full length *xopN* gene from genomic DNA of wild type strain of Xoo cloned into *Sma*I site of p*Ubi*-C1300; Km^r^	This work

^a^ Km^r^ and Sp^r^ indicate resistance to kanamycin and spectinomycin respectively. Xoo implies *X* *. oryzae* pv. *oryzae* .

**Table 4:** Plasmids used in this study.

**Plasmids**	**Relevant characteristics^a^**	**Reference/Source**
p*Ubi*-*xopW*	p*Ubi*-C1300 with 624-bp fragment containing full length *xopW* gene from genomic DNA of wild type strain of Xoo cloned into *Kpn*I and *Sac*I site of p*Ubi*-C1300; Km^r^	This work
p*Ubi*-*xopY*	p*Ubi*-C1300 with 848-bp fragment containing full length *xopY* gene from genomic DNA of wild type strain of Xoo cloned into *Kpn*I and *Sac*I site of p*Ubi*-C1300; Km^r^	This work
p*Ubi*-*xopT*	p*Ubi*-C1300 with 964-bp fragment containing full length *xopT* gene from genomic DNA of wild type strain of Xoo cloned into *Kpn*I and *Sac*I site of p*Ubi*-C1300; Km^r^	This work
p*Ubi*-*xopAA*	p*Ubi*-C1300 with 1746-bp fragment containing full length *xopAA* gene from genomic DNA of wild type strain of Xoo cloned into *Kpn*I and *Sac*I site of p*Ubi*-C1300; Km^r^	This work
p*Ubi*-*xopAB*	p*Ubi*-C1300 with 623-bp fragment containing full length *xopAB* gene from genomic DNA of wild type strain of Xoo cloned into *Kpn*I and *Sac*I site of p*Ubi*-C1300; Km^r^	This work
p*Ubi*-*xopP*	p*Ubi*-C1300 with 2194-bp fragment containing full length *xopP* gene from genomic DNA of wild type strain of Xoo cloned into *Sma*I site of p*Ubi*-C1300; Km^r^	This work
pENTR-*avrBs2*	pENTR/D/TOPO with 708-bp fragment containing full length *avrBs2* gene from genomic DNA of wild type strain of Xoo; Km^r^	This work
pENTR-*xopK*	pENTR/D/TOPO with 2538-bp fragment containing full length *xopK* gene from genomic DNA of wild type strain of Xoo; Km^r^	This work
pENTR-*xopZ*	pENTR/D/TOPO with 3867-bp fragment containing full length *xopZ* gene from genomic DNA of wild type strain of Xoo; Km^r^	This work
pENTR-*xopU*	pENTR/D/TOPO with 2958-bp fragment containing full length *xopU* gene from genomic DNA of wild type strain of Xoo; Km^r^	This work
pENTR-*xopV*	pENTR/D/TOPO with 996-bp fragment containing full length *xopV* gene from genomic DNA of wild type strain of Xoo; Km^r^	This work
pENTR-*xopX*	pENTR/D/TOPO with 2190-bp fragment containing full length *xopX* gene from genomic DNA of wild type strain of Xoo; Km^r^	This work
pENTR-*xopR*	pENTR/D/TOPO with 1314-bp fragment containing full length *xopR* gene from genomic DNA of wild type strain of Xoo; Km^r^	This work

^a^ Km^r^ and Sp^r^ indicate resistance to kanamycin and spectinomycin respectively. Xoo implies *X* *. oryzae* pv. *oryzae* .

**Table 5:** Plasmids used in this study.

**Plasmids**	**Relevant characteristics^a^**	**Reference/Source**
pENTR-*xopQ*	pENTR/D/TOPO with 1392-bp fragment containing full length *xopQ* gene from genomic DNA of wild type strain of Xoo; Km^r^	This work
pENTR-*xopF*	pENTR/D/TOPO with 1986-bp fragment containing full length *xopF* gene from genomic DNA of wild type strain of Xoo; Km^r^	This work
pMDC7-*avrBs2*	pMDC7 with 708-bp fragment containing full length *avrBs2* gene obtained from recombination of pENTR-*avrBs2* with pMDC7; Sp^r^	This work
pMDC7-*xopK*	pMDC7 with 2538-bp fragment containing full length *xopK* gene obtained from recombination of pENTR-*xopK* with pMDC7; Sp^r^	This work
pMDC7-*xopV*	pMDC7 with 996-bp fragment containing full length *xopV* gene obtained from recombination of pENTR-*xopV* with pMDC7; Sp^r^	This work
pMDC7-*xopZ*	pMDC7 with 3867-bp fragment containing full length *xopZ* gene obtained from recombination of pENTR-*xopZ* with pMDC7; Sp^r^	This work
pMDC7-*xopX*	pMDC7 with 2190-bp fragment containing full length *xopX* gene obtained from recombination of pENTR-*xopX* with pMDC7; Sp^r^	This work
pMDC7-*xopR*	pMDC7 with 1314-bp fragment containing full length *xopR* gene obtained from recombination of pENTR-*xopR* with pMDC7; Sp^r^	This work
pMDC7-*xopQ*	pMDC7 with 1392-bp fragment containing full length *xopQ* gene obtained from recombination of pENTR-*xopQ* with pMDC7; Sp^r^	This work
pMDC7-*xopU*	pMDC7 with 2958-bp fragment containing full length *xopU* gene obtained from recombination of pENTR-*xopU* with pMDC7; Sp^r^	This work
pMDC7-*xopF*	pMDC7 with 1986-bp fragment containing full length *xopF* gene obtained from recombination of pENTR-*xopF* with pMDC7; Sp^r^	This work

^a^ Km^r^ and Sp^r^ indicate resistance to kanamycin and spectinomycin respectively. Xoo implies *X* *. oryzae* pv. *oryzae* .

### Molecular biological techniques

Genomic DNA was isolated as described by Leach and co-workers [42]. Plasmid DNA was isolated by using either a Qiagen (Hilden, Germany) plasmid midi-kit or alkaline lysis method [43]. High-fidelity Phusion polymerase from Finnzymes (Thermo scientific, Rockford, IL, USA) was used for PCR amplification of insert DNA for all cloning purposes while Taq polymerase was used for all other applications. Restriction digestions were done with enzymes from New England Biolabs (NEB, Beverly, MA, USA). PCR products and restriction enzyme-digested DNA fragments were purified using a QIAquick PCR purification kit and a QIAquick nucleotide removal kit (Qiagen), respectively. Ligation with T4 DNA ligase, agarose gel electrophoresis, and transformation of *E. coli* were performed as described previously [43].

### DNA sequencing and bioinformatics tools

DNA sequencing was done with an ABI Prism 3700 automated DNA sequencer (PerkinElmer, Foster City, CA, USA). BLAST algorithm in the NCBI database [44] was used for homology and conserved domain searches. No new DNA sequence has been generated in this study.

### Construction and complementation of xopN**^-^**, xopQ**^-^**, xopX**^-^** and xopZ**^-^**, mutants of X. oryzae pv. oryzae

A *xopN* ^*-*^ mutant of *X* *. oryzae* pv. *oryzae* was generated by gene disruption using homologous plasmid integration of a suicide vector pK18*mob* [45]. A 497-bp internal fragment of the *xopN* gene was amplified by PCR using genomic DNA of *X* *. oryzae* pv. *oryzae* wild type strain and the gene-specific XopN1F/XopN1R (Table S1) oligonucleotide primer pair. This fragment was cloned into the *Sma*I digested blunt-ended pK18*mob* cloning vector to obtain recombinant plasmid pDS1. The plasmid pDS1 was then electroporated into wild type and clones were selected for kanamycin resistance to obtain a *xopN* ^-^ mutant by plasmid integration. Gene disruption was confirmed by PCR using *xopN* gene-specific flanking primers XopNF/XopNR in combination with vector-specific primers M13F and M13R and sequencing of PCR products. The *xopN* gene fragment was cloned in the same transcriptional orientation as the *lacZ* promoter of pK18*mob* vector in pDS1 so that mutation caused by pDS1 integration does not have a polar effect on downstream genes due to activity of the outwardly directed *lacZ* promoter [46,47].

For *xopN* ^*-*^ mutant complementation, a 2225-bp DNA fragment containing the full length of *xopN* gene was amplified by PCR using primers XopN2F/XopN2R and total genomic DNA of *X* *. oryzae* pv. *oryzae* as template. The amplified DNA fragment was cloned as a *Hind*III-*Sac*I fragment into the broad host range vector pHM1 [48] to create the recombinant plasmid pDS2. The pDS2 construct and the pHM1 vector were individually introduced into the *xopN* ^*-*^mutant strain by electroporation and selection for spectinomycin resistance. The presence of pDS2 was confirmed by PCR using *xopN* gene-specific flanking primers XopN2F/XopN2R and universal primers M13F/M13R and sequencing of PCR products. The confirmed clones were designated as *xopN* ^*-*^ */xopN* ^*+*^ (*xopN* ^*-*^/pDS2; for complementation) and *xopN* ^*-*^ */*pHM1 (*xopN* ^*-*^/pHM1; vector control) and studied further.

A *xopX* ^*-*^ mutant of *X* *. oryzae* pv. *oryzae* was isolated by creating an inframe deletion in the *xopX* gene. For this purpose, 729 and 732 bp fragments of the *xopX* gene were amplified by PCR using genomic DNA of wild type strain and the gene-specific XopXS1F/XopXS1R and XopXS3F/XopXS3R primer pairs, respectively. The primers XopXS1F and XopXS3R were having an engineered site for restriction enzyme *Hind*III at their 5’ and 3’ ends, respectively. Similarly, the primers XopXS1R and XopXS3F were having an engineered site for restriction enzyme *Xba*I at their 3’ and 5’ ends, respectively. The amplified fragments were digested with *Xba*I restriction enzyme. The *Xba*I-digested 729 and 732 bp fragments were ligated and the ligated product was PCR amplified using the gene-specific XopXS1F/XopXS3R primer pair. A 1473 bp fragment was obtained which included a 12 bp insertion that contains the *Xba*I site. The 1461bp fragment, which now contained *Hin*dIII sites at the 5’ and 3’ ends, was digested with *Hin*dIII restriction enzyme and cloned into the *Hin*dIII site of the pK18*mobsac*B cloning vector to obtain the recombinant plasmid pDS3. Sequencing of pDS3 confirmed that no other changes had occurred in the target *xopX* sequence except an inframe deletion of 729 bp (243 aa) and insertion of 12 bp (*Xba*I restriction enzyme site along with adapter sequences). This construct was then transferred from *E. coli* S17-1 to wild type via biparental mating. The transformants were plated on nutrient agar (Difco, Detroit, MI, USA) plates containing Km. Single recombinant colonies that were kanamycin-resistant and sucrose-sensitive were grown and passaged thrice in nutrient broth without Km and sucrose. Saturated bacterial cultures were dilution plated on PSA plates containing 1% sucrose to obtain double recombinant strains that were sucrose-resistant and kanamycin-sensitive. The inframe deletion that had been incorporated into the *xopX* gene on the *X* *. oryzae* pv. *oryzae* chromosome was confirmed by PCR using *xopX* gene-specific flanking primers XopXS1F/XopXS3R and sequencing of PCR products. One of the confirmed mutant clones was designated *xopX* ^*-*^ mutant and chosen for further study. Using a similar strategy, *xopQ* ^*-*^ and *xopZ* ^*-*^ mutants of *X* *. oryzae* pv. *oryzae* were isolated by creating inframe deletions in the *xopQ* and *xopZ* genes. Briefly, 420 and 450 bp fragments of the *xopQ* gene were PCR amplified using genomic DNA of wild type strain and the gene-specific XopQS1F/XopQS1R and XopQS3F/XopQS3R primer pairs, respectively. These PCR amplified fragments were then ligated as *Xba*I-digested fragments. Similarly, 1304 and 1254 bp PCR fragments of the *xopZ* gene were obtained using genomic DNA of wild type strain and the gene specific XopZS1F/XopZS1R and XopZS3F/XopZS3R primer pairs, respectively. These PCR amplified fragments were digested with *Xba*I and ligated to each other. The corresponding ligated products of *xopQ* and *xopZ* genes were PCR amplified as 882 and 2570 bp fragments which included a 12 bp insertion that contains the *Xba*I site, using XopQS1F/XopQS3R and XopZS1F/XopZS3R primer pairs, respectively. These fragments were digested with *Hind*III and cloned individually into the *Hind*III site of pK18*mobsac*B to obtain the recombinant plasmids pDS5 and pDS7, respectively. The constructs pDS5 and pDS7 were confirmed to carry no other changes in the target *xopQ* and *xopZ* gene sequences except inframe deletions of 513 bp (171 aa) and 1293 bp (431 aa), respectively, and insertions of 12 bp (*Xba*I restriction enzyme site along with adapter sequences). These constructs were then transferred from *E. coli* S17-1 to wild type via biparental mating and marker free *xopQ* ^*-*^ and *xopZ* ^*-*^ mutants were generated following the steps as described above for generation of *xopX* ^*-*^ mutant strain. The inframe deletions that had been incorporated into the *xopQ* and *xopZ* genes on the *X* *. oryzae* pv. *oryzae* chromosome were confirmed by PCR using *xopQ* and *xopZ* gene-specific flanking primers XopQS1F/XopQS3R and XopZS1F/XopZS3R respectively, and sequencing of PCR products. The mutants for *xopQ* and *xopZ* genes were designated as *xopQ* ^*-*^ and *xopZ* ^*-*^, respectively.

For *xopX* ^*-*^ mutant complementation, a 2190 bp DNA fragment containing the full length of *xopX* gene was amplified by PCR using primers XopX2F/XopX2R and total genomic DNA of *X* *. oryzae* pv. *oryzae* as template. The amplified DNA fragment was cloned as a *Hind*III-*Sac*I fragment into the broad host range vector pHM1 [48] to create the recombinant plasmid pDS4. The pDS4 plasmid and pHM1 vector were individually introduced into the *xopX* ^*-*^ mutant strain by electroporation and the recombinants were isolated by selection for spectinomycin resistance. The presence of pDS4 was confirmed by PCR using *xopX* gene-specific flanking primers XopX2F/XopX2R and universal primers M13F/M13R and sequencing of PCR products. The confirmed clones were designated as *xopX* ^*-*^ */xopX* ^*+*^ (*xopX* ^*-*^/pDS4; for complementation) and *xopX* ^*-*^ */*pHM1 (*xopX* ^*-*^/pHM1; vector control) and studied further. A similar strategy was used for complementation of *xopQ* ^*-*^ and *xopZ* ^*-*^ mutants. Briefly, the full length copies of *xopQ* and *xopZ* genes were PCR amplified from *X* *. oryzae* pv. *oryzae* genomic DNA using primer pairs, XopQ2F/XopQ2R and XopZ2F/XopZ2R, respectively. PCR fragments of *xopQ* and *xopZ* genes were individually cloned as HindIII-SacI fragment in vector pHM1 to create the constructs pDS6 and pDS8, respectively. The pDS6 and pDS8 constructs were individually introduced by electroporation into the *xopQ* ^-^ mutant and *xopZ* ^-^ mutants, respectively, to get spectinomycin resistant recombinant clones. The presence of pDS6 and pDS8 was confirmed by PCR using respective gene-specific flanking primers XopQ2F/XopQ2R and XopZ2F/XopZ2R, and universal primers M13F/M13R and sequencing of PCR products. The confirmed clones for complementation of *xopQ* ^*-*^ and *xopZ* ^-^ mutants were designated as *xopQ* ^*-*^ */xopQ* ^*+*^ (*xopQ* ^*-*^/pDS6) and *xopZ* ^*-*^ */xopZ* ^*+*^ (*xopZ* ^*-*^/pDS8) respectively. For vector control, pHM1 vector was individually introduced by electroporation into the *xopQ* ^*-*^ mutant and the *xopZ* ^-^ mutant. Spectinomycin resistant clones were designated as *xopQ* ^-^ /pHM1 (vector control) and *xopZ* ^-^ /pHM1 (vector control) and used for further study.

### Construction of xopN**^-^** xopX**^-^** double and xopN**^-^** xopQ**^-^** xopX**^-^** xopZ**^-^** quadruple mutants of X. oryzae pv. oryzae

A *xopN* ^*-*^ *xopX* ^-^ double mutant of *X* *. oryzae* pv. *oryzae* was isolated by gene disruption of *xopN* using homologous plasmid integration in a *xopX* ^*-*^ mutant strain. The construct pDS1 was electroporated into electrocompetent cells of *xopX* ^*-*^ mutant strain to obtain a *xopN* ^*-*^ *xopX* ^-^ double mutant by plasmid integration. The *xopN* gene disruption in *xopN* ^*-*^ *xopX* ^-^ mutant strain was confirmed as described above for the mutation in *xopN* ^*-*^ strain.

A *xopQ* ^*-*^ *xopX* ^*-*^ double mutant of *X* *. oryzae* pv. *oryzae* was isolated by creating an inframe deletion of 513 bp (171 aa) in the *xopQ* gene in the genome of *xopX* ^-^ mutant strain. For this purpose, the construct pDS5 was transferred from *E. coli* S17-1 to *xopX* ^-^ mutant via biparental mating. An inframe deletion was created in the *xopQ* gene in the genetic background of the *xopX* ^-^ mutant. One of the confirmed clones was designated as *xopQ* ^*-*^ *xopX* ^*-*^ double mutant strain.

In a similar way, a *xopQ* ^*-*^ *xopX* ^*-*^ *xopZ* ^*-*^ triple mutant of *X* *. oryzae* pv. *oryzae* was generated by incorporating an inframe deletion of 1293 bp (431 aa) in the *xopZ* gene in the genome of *xopQ* ^*-*^ *xopX* ^*-*^ double mutant strain. To achieve this, biparental mating was set up to mobilize the construct pDS7 from *E. coli* S17-1 to *xopQ* ^*-*^ *xopX* ^*-*^ double mutant. The inframe deletion that had been incorporated in the *xopZ* gene in the genomic background of *xopQ* ^*-*^ *xopX* ^*-*^ double mutant strain was confirmed as described earlier for isolating the *xopZ* ^*-*^ mutant strain. One of the confirmed clones was designated as a *xopQ* ^*-*^ *xopX* ^*-*^ *xopZ* ^*-*^ triple mutant of *X* *. oryzae* pv. *oryzae* . A *xopN* ^*-*^ *xopQ* ^*-*^ *xopX* ^*-*^ *xopZ* ^*-*^ quadruple mutant of *X* *. oryzae* pv. *oryzae* was isolated by disrupting the *xopN* gene using homologous plasmid integration in the *xopZ* ^*-*^ *xopQ* ^*-*^ *xopX* ^*-*^ triple mutant strain. A disruption of the *xopN* gene was achieved by electroporating the construct pDS1 into *xopQ* ^*-*^ *xopX* ^*-*^ *xopZ* ^*-*^ triple mutant strain and selecting for kanamycin resistant transformants. Generation of the *xopN* ^*-*^ mutant was confirmed by PCR using *xopN* gene-specific flanking primers XopNF/XopNR in combination with vector-specific primers M13F and M13R and sequencing the PCR products. One of the confirmed clones was designated as *xopN* ^*-*^ *xopQ* ^*-*^ *xopX* ^*-*^ *xopZ* ^*-*^ quadruple mutant of *X* *. oryzae* pv. *oryzae* . For complementation of *xopN* ^*-*^ *xopQ* ^*-*^ *xopX* ^*-*^ *xopZ* ^*-*^ quadruple mutant with *xopN* gene, the pDS2 plasmid was introduced into the *xopN* ^*-*^ *xopQ* ^*-*^ *xopX* ^*-*^ *xopZ* ^*-*^ quadruple mutant by electroporation and selection of spectinomycin resistant colonies. The presence of pDS2 was confirmed by PCR. As a control, the pHM1 vector was introduced into the *xopN* ^*-*^ *xopQ* ^*-*^ *xopX* ^*-*^ *xopZ* ^*-*^ quadruple mutant in a similar manner.

### Cell death assay

Surface sterilized rice (Taichung Native-1; a susceptible rice variety) seeds were germinated on 0.5% sterile agar in Petri dishes for 2 to 3 days. Root tips, 1 to 2 cm long, were excised from the seedlings and treated with 200 µl of either purified LipA (500 µg/ml) or buffer (10 mM phosphate buffer, pH 6.0). After incubation for 18-20 h, roots were washed and stained with propidium iodide (PI) for 20 min and mounted in 50% glycerol on glass slides. The samples were viewed under a LSM-510 Meta confocal microscope (Carl Zeiss, Germany) using 63 × oil immersion objectives and He-Ne laser at 543 nm excitation to detect PI internalization. Longitudinal optical sections of 1.0 µm thickness were acquired and further projected to obtain the image of 2 to 3 µm total thickness. All images were analyzed using LSM software and further edited using Photoshop (Adobe, San Jose, CA, USA).

### Preinoculation with *Agrobacterium tumefaciens* EHA105 strains or *X* *. oryzae* pv. *oryzae* strains

*X* *. oryzae* pv. *oryzae* strains were grown in PS broth to saturation, pelleted, washed with buffer (10 mM phosphate buffer, pH 6.0) and resuspended in buffer. The cells from saturated cultures of *Agrobacterium* strains grown in TYM broth were pelleted, washed following centrifugation and resuspended in induction medium (10 mM MES, pH 5.6, 10 mM MgCl_2,_ and 150 µM acetosyringone; Sigma) and then incubated in a shaker for 2 h at 28°C [8]. Rice roots were pretreated with either suspensions of *Agrobacterium* strains for 4-5 h in induction media, in the presence or absence of 17-β-estradiol (20 µM final concentration), or *X* *. oryzae* pv. *oryzae* strains dissolved in phosphate buffer for 2 h at 28°C. After rinsing with fresh buffer, the roots were treated with purified LipA (~500 µg/ml) for 18-20 h at 28°C and processed further for confocal analyses as described above. As controls, rice roots pretreated with either *Agrobacterium* strains or *X* *. oryzae* pv. *oryzae* strains and subsequently treated with buffer for 18-20 h at 28°C were also analysed by confocal microscopy. All the bacterial strains were used at a final concentration of OD=1.5. At least 200 µl volumes of either of LipA, buffer or culture were used to treat the rice roots.

### Callose deposition assay

Leaves of 10-day old rice seedlings were infiltrated using the blunt end of a 1 ml syringe with one of the following : buffer, water, purified LipA (~100 µg/ml) and saturated culture of *X* *. oryzae* pv. *oryzae* T3S^-^ mutant strain. Bacteria were collected from saturated cultures of *Agrobacterium* strains, washed and resuspended in induction medium (10 mM MES, pH 5.6, 10 mM MgCl_2_ and 150 µM acetosyringone) and then incubated in a shaker for 2 h at 28°C before infiltration [8]. *Agrobacterium* strains were recollected from induction medium by centrifugation and processed further. Rice leaves were coinfiltrated with *Agrobacterium* strains resuspended in buffer containing purified LipA (~100 µg/ml). *Agrobacterium* strains containing the pMDC7 vector were resuspended in purified LipA (~100 µg/ml) with and without 17-β-estradiol (20 µM final concentration). All the bacterial strains ( *X* *. oryzae* pv. *oryzae* and *Agrobacterium* ) were used to final concentration of OD=1.0. After 20-24 h, the infiltrated zones (~1× 1 cm) were cut from the leaves, destained with 70% ethanol at 65°C, stained with 0.5% aniline blue for 4 h and observed under an Axioplan2 epifluorescence microscope (Carl Zeiss, Germany) using a blue filter (excitation wavelength of 365 nm) and ×10 objective [49].

### Virulence assay

For virulence analysis various *X* *. oryzae* pv. *oryzae* strains were grown to saturation in PS broth supplemented with appropriate antibiotics. The cells were pelleted down by centrifugation, washed and resuspended in sterile water at a concentration of OD=1.0. Surgical scissors dipped in these bacterial suspensions were used to clip the leaf tips of greenhouse-grown, 40-day-old plants of the susceptible rice variety Taichung Native-1 (TN-1) [50]. Lesion lengths were measured 7 days after inoculation.

### Cloning of T3S effectors of *X* *. oryzae* pv. *oryzae* into *Agrobacterial* T-DNA vectors

Full length *xopN* and *xopP* genes were amplified by PCR using genomic DNA of wild type strain of *X* *. oryzae* pv. *oryzae* and the corresponding gene-specific oligoneucletide primer pair (Table S1) and cloned into the *Sma*I site of the binary vector p*Ubi*-C1300 [26] between the maize *ubiquitin* promoter and *nos3*’ terminator to obtain recombinant plasmids p*Ubi*-*xopN* and p*Ubi*-*xopP* respectively. Full length *xopW*, *xopY*, *xopT*, *xopAA* and *xopAB* genes were PCR amplified using genomic DNA of wild type *X* *. oryzae* pv. *oryzae* strain and the corresponding gene-specific oligonucleotide primer pairs (Table S1). The PCR products were cloned individually as *Kpn*I-*Sac*I fragments into the binary vector p*Ubi*-C1300 to obtain the recombinant plasmids p*Ubi*-*xopW*, p*Ubi*-*xopY*, p*Ubi*-*xopT*, p*Ubi*-*xopAA* and p*Ubi*-*xopAB* respectively. Positive clones were screened using gene-specific and vector-specific primers and confirmed by sequencing. The full length genes of *xopR*, *xopV*, *xopK*, *xopF*, *xopQ*, *xopU*, *xopX*, *xopZ* and *avrBs2* were PCR amplified from genomic DNA of wild type *X* *. oryzae* pv. *oryzae* strain with engineered 5’ CACC overhang were cloned individually into cloning vector pENTR/D/TOPO (Invitrogen, Carlsbad, CA, USA) to obtain plasmids pENTR-*xopR*, pENTR-*xopV*, pENTR-*xopK*, pENTR-*xopF*, pENTR-*xopQ*, pENTR-*xopU*, pENTR-*xopX*, pENTR-*xopZ* and pENTR-*avrBs2* respectively. Positive clones in *E. coli* DH5α were screened using gene-specific primers and confirmed by sequencing. Plasmids from these clones were isolated and recombined into the 17-β-estradiol inducible binary cloning vector pMDC7 [51] by Gateway LR reaction (Invitrogen) to obtain recombinant plasmids pMDC7-*xopR*, pMDC7-*xopV*, pMDC7-*xopK*, pMDC7-*xopF*, pMDC7-*xopQ*, pMDC7-*xopU*, pMDC7-*xopX*, pMDC7-*xopZ* and pMDC7-*avrBs2* respectively. Clones obtained in *E. coli* DH5α were screened using PCR with appropriate primer pairs and confirmed by sequencing. Plasmids containing cloned genes in the binary vectors (either p*Ubi*-C1300 or pMDC7) were isolated and further electroporated into electrocompetent cells of *A. tumefaciens* strain EHA105.

### RT-PCR analysis to determine expression of *xop* genes of *X* *. oryzae* pv. *oryzae* in rice roots

Rice roots were treated with *Agrobacterium* EHA105 strains carrying any one of *xopN*, *xopP, xopQ, xopR, xopX* or *xopZ* genes on the T-DNA vector. The *xopQ, xopR, xopX* and *xopZ* genes are cloned in a T-DNA vector, pMDC7, wherein the expression of the cloned gene is under the induction of 17-β-Estradiol (Est). The *xopN* and *xopP* is cloned in T-DNA vector p*Ubi*-C1300, wherein its expression is under the control of maize ubiquitin promoter. Accordingly, as negative controls, for the genes cloned in Est inducible vector (pMDC7), rice roots were treated with Agrobacterial strains in the absence of Est, whereas for genes cloned in vector p*Ubi*-C1300, rice roots were treated with *Agrobacterium* strain containing an empty vector. Total RNA was isolated from rice tissue treated with different *Agrobacterium* EHA105 strains (with or without Est wherever needed) using Trizol reagent (Invitrogen) according to the manufacturer’s instructions. The quality of isolated RNA was assessed by agarose gel electrophoresis and quantitated using spectrophotometer (Nanodrop ND-1000, Thermo Scientific). 5 µg of total RNA samples were treated with RNasefree DNaseI (Promega) for 1 h at 37°C to remove any DNA contamination. After inactivation of DNaseI, 1 µg RNA was used for cDNA synthesis using SuperscriptIII (Invitrogen) RT enzyme as per the manufacturer’s instructions and oligo (dT) primers. Subsequently, each cDNA was used as template for PCR amplification with Taq polymerase using gene-specific primers (Table S2) designed to amplify fragments of each gene of interest. The DNaseI treated RNA that was not reverse transcribed was used as template in PCR as negative control to show that the RT-PCR products obtained were not because of any DNA contamination in the cDNA samples. The amplification condition used was 95°C for 5 min followed by 28 cycles of 94°C for 30 sec, 58°C for 30 sec, 72°C for 45 sec, again followed by 72°C for 10 min. The RT-PCR amplified products were analysed by agarose gel electrophoresis and staining with ethidium bromide.

## Supporting Information

Figure S1 **Expression of *xopN, xopP xopQ*, *xopR, xopX* and *xopZ* genes of *Xanthomonas* *oryzae* pv. *oryzae* in rice roots following *Agrobacterium* mediated transient transfer.** cDNA was synthesised from total RNA isolated from rice roots treated with one of the following: EHA105/p*Ub*i-*xopN*; EHA105/p*Ub*i-*xopP*; EHA105/p*Ub*i (empty vector); EHA105/pMDC7-*xopQ* with Estradiol (Est) or without Est; EHA105/pMDC7-*xopR* with Est or without Est; EHA105/pMDC7-*xopX* with Est or without Est; EHA105/pMDC7-*xopZ* with Est or without Est. Subsequently, each cDNA was used as a template for PCR amplification with Taq polymerase using gene-specific primers (Table S2) designed to amplify a 118-164 bp fragment of each gene of interest. (**A**) PCR products of 123 bp (XopNRTF/XopNRTR primer pair) and 164 bp (XopPRTF/XopPRTR primer pair) were obtained using, as a template, cDNA prepared with RNA from roots treated with EHA105/p*Ub*i-*xopN* and EHA105/p*Ub*i-*xopP*, respectively. These products were not obtained when cDNA prepared from roots treated with EHA105/p*Ub*i was used as a template. PCR product of 158 bp (GAPDHF/GAPDHR primer pair which amplifies a fragment from the rice GAPDH gene) was obtained using cDNA prepared from roots treated with any of the three Agrobacterial strains. (**B**) PCR products of 148 bp (XopQRTF/XopQRTF primer pair), 128 bp (XopRRTF/XopRRTF primer pair), 143 bp (XopXRTF/XopXRTF primer pair) and 149 bp (XopZRTF/XopZRTF primer pair) were obtained using, as a template, cDNA prepared with RNA from roots treated with EHA105/pMDC7-*xopQ*, EHA105/pMDC7-*xopR*, EHA105/pMDC7-*xopX* and EHA105/pMDC7-*xopZ* in the presence of Est but not in the absence of Est, respectively. A PCR product of 158 bp (GAPDHF/GAPDHR primer pair) was obtained using, as a template, cDNA obtained from roots treated with any of the above vectors either in the presence or absence of estradiol.
(TIF)

Figure S2 **A *xopN*^*-*^* xopQ*^*-*^* xopX*^*-*^* xopZ*^*-*^ quadruple mutant of *Xanthomonas* *oryzae* pv.*oryzae* induces PCD in rice roots.** Rice roots were treated with one of the following: wild type *X* *. oryzae* pv. *oryzae* (**A**); *xopN* ^*-*^ *xopQ* ^*-*^ *xopX* ^*-*^ *xopZ* ^*-*^ quadruple mutant (**B**); T3S^-^ mutant (**C**). Treated roots were subsequently stained with propidium iodide (PI) and viewed under a confocal microscope. Internalisation of PI is indicative of defense response-associated programmed cell death in rice roots. Scale bar measures 20µm.
(TIF)

Figure S3 **Complementation with the *xopN* gene reduces ability of the *xopN*^*-*^* xopZ*^*-*^* xopQ*^*-*^* xopX*^*-*^ quadruple mutant to induce callose deposition in rice leaves.** Rice leaves were infiltrated with one of the following: *xopQ* ^*-*^ *xopX* ^*-*^ *xopZ* ^*-*^ triple mutant, *xopN* ^*-*^ *xopQ* ^*-*^ *xopX* ^*-*^ *xopZ* ^*-*^ quadruple mutant, *xopN* ^*-*^ *xopQ* ^*-*^ *xopX* ^*-*^ *xopZ* ^*-*^ /*xopN* ^*+*^ (quadruple mutant complemented with *xopN* gene) and *xopN* ^*-*^ *xopQ* ^*-*^ *xopX* ^*-*^ *xopZ* ^*-*^/pHM1 (quadruple mutant with pHM1 plasmid; vector control). The leaves were subsequently stained with aniline blue and visualized under an epifluorescence microscope. Callose deposits were quantified from 0.60 mm^2^ area of an infiltrated leaf. Data were collected from atleast five leaves in each experiment (three experiments indicated as ExpI, ExpII and ExpIII) and 2-3 different viewing areas from the infiltrated region of each leaf. Statistically significant differences at *P* < 0.05 (Student’s two-tailed *t* test for independent means) were obtained from leaves infiltrated with either *xopN* ^*-*^ *xopQ* ^*-*^ *xopX* ^*-*^ *xopZ-*/*xopN* ^*+*^ or *xopQ* ^*-*^ *xopX* ^*-*^ *xopZ* ^*-*^ triple mutant as compared to leaves treated with a *xopN* ^*-*^ *xopQ* ^*-*^ *xopX* ^*-*^ *xopZ* ^*-*^ quadruple mutant. Statistically significant differences were not observed in the following comparisons: either *xopN* ^*-*^ *xopQ* ^*-*^ *xopX* ^*-*^ *xopZ-*/*xopN* ^*+*^ with *xopQ* ^*-*^ *xopX* ^*-*^ *xopZ* ^*-*^ triple mutant or *xopN* ^*-*^ *xopQ* ^*-*^ *xopX* ^*-*^ *xopZ* ^*-*^ with *xopN* ^*-*^ *xopZ* ^*-*^ *xopQ* ^*-*^ *xopX* ^*-*^/pHM1.
(TIF)

Figure S4 ***xopN*^*-*^, *xopQ*^*-*^, *xopX*^*-*^ or *xopZ*^*-*^* single mutants* of *Xanthomonas* *oryzae* pv. *oryzae* induce callose deposition at a basal level.** Rice leaves were infiltrated with one of the following: wild type *X* *. oryzae* pv. *oryzae* , *xopN* ^*-*^ mutant, *xopQ* ^*-*^ mutant, *xopX* ^*-*^ mutant, *xopZ* ^*-*^ mutant. The leaves were subsequently stained with aniline blue and visualized under an epifluorescence microscope. Callose deposits were quantified from 0.60 mm^2^ area of an infiltrated leaf. Data were collected from atleast five leaves in each experiment and 2-3 different viewing areas from the infiltrated region of each leaf. Data from one experiment are represented. Similar results were obtained in independent experiments.
(TIF)

Table S1 **List of oligonucleotide primers used in this study.**
(DOCX)

Table S2 **List of oligonucleotide primers used for RT-PCR.**
(DOCX)
