# Supplementary figures and images for: Cell Wall Degrading Enzyme Induced Rice Innate Immune Responses Are Suppressed by the Type 3 Secretion System Effectors XopN, XopQ, XopX and XopZ of Xanthomonas oryzae pv. oryzae
Source: PLoS One. 2013 Sep 26;8(9):e75867. doi: 10.1371/journal.pone.0075867 (PMC3784402; doi:10.1371/journal.pone.0075867)

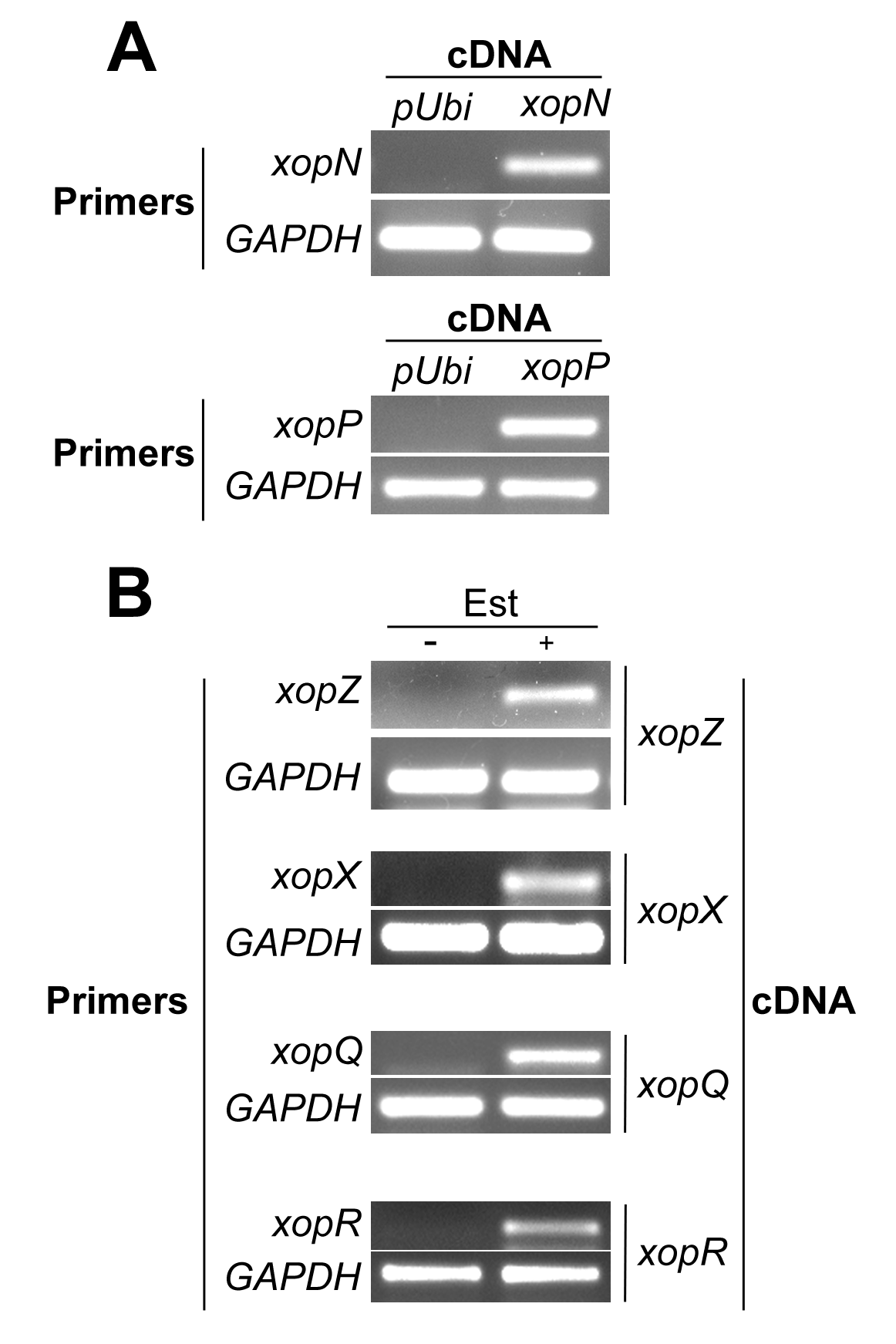

Supplement: Figure S1 — Expression of xopN, xopP xopQ, xopR, xopX and xopZ genes of Xanthomonas oryzae pv. oryzae in rice roots following Agrobacterium mediated transient transfer. cDNA was synthesised from total RNA isolated from rice roots treated with one of the following: EHA105/pUbi-xopN; EHA105/pUbi-xopP; EHA105/pUbi (empty vector); EHA105/pMDC7-xopQ with Estradiol (Est) or without Est; EHA105/pMDC7-xopR with Est or without Est; EHA105/pMDC7-xopX with Est or without Est; EHA105/pMDC7-xopZ with Est or without Est. Subsequently, each cDNA was used as a template for PCR amplification with Taq polymerase using gene-specific primers (Table S2) designed to amplify a 118-164 bp fragment of each gene of interest. (A) PCR products of 123 bp (XopNRTF/XopNRTR primer pair) and 164 bp (XopPRTF/XopPRTR primer pair) were obtained using, as a template, cDNA prepared with RNA from roots treated with EHA105/pUbi-xopN and EHA105/pUbi-xopP, respectively. These products were not obtained when cDNA prepared from roots treated with EHA105/pUbi was used as a template. PCR product of 158 bp (GAPDHF/GAPDHR primer pair which amplifies a fragment from the rice GAPDH gene) was obtained using cDNA prepared from roots treated with any of the three Agrobacterial strains. (B) PCR products of 148 bp (XopQRTF/XopQRTF primer pair), 128 bp (XopRRTF/XopRRTF primer pair), 143 bp (XopXRTF/XopXRTF primer pair) and 149 bp (XopZRTF/XopZRTF primer pair) were obtained using, as a template, cDNA prepared with RNA from roots treated with EHA105/pMDC7-xopQ, EHA105/pMDC7-xopR, EHA105/pMDC7-xopX and EHA105/pMDC7-xopZ in the presence of Est but not in the absence of Est, respectively. A PCR product of 158 bp (GAPDHF/GAPDHR primer pair) was obtained using, as a template, cDNA obtained from roots treated with any of the above vectors either in the presence or absence of estradiol. (TIF) [file pone.0075867.s001.tif]

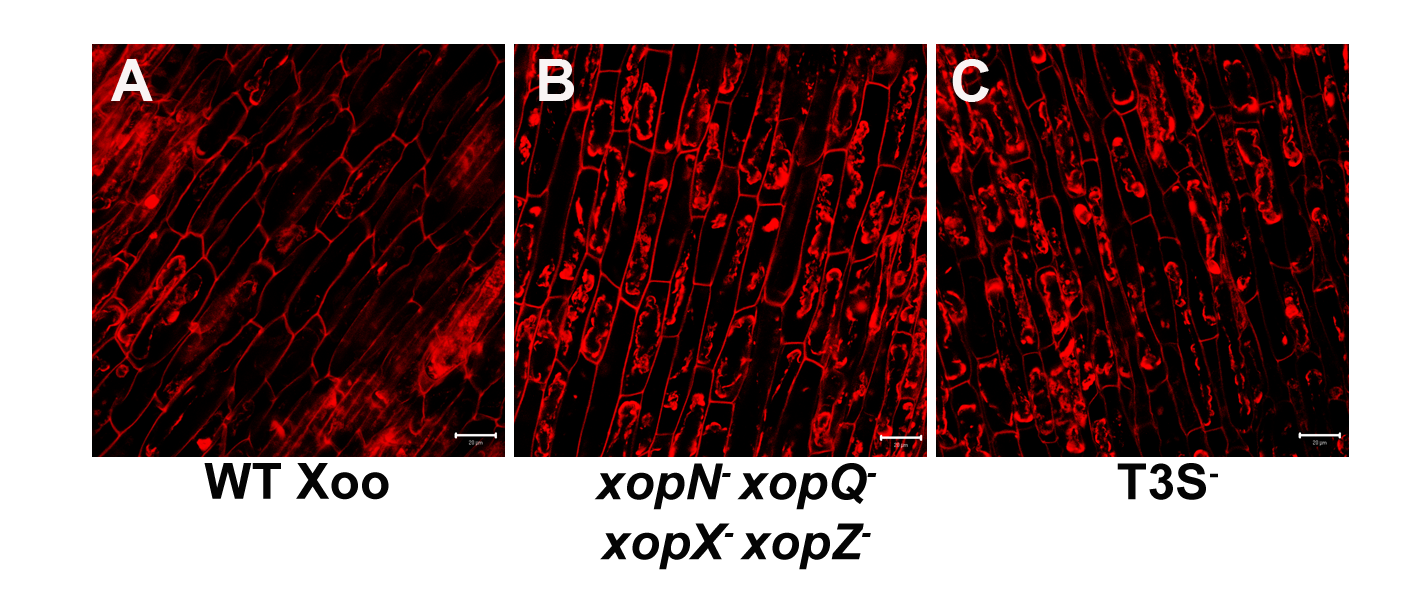

Supplement: Figure S2 — A xopN- xopQ- xopX- xopZ- quadruple mutant of Xanthomonas oryzae pv.oryzae induces PCD in rice roots. Rice roots were treated with one of the following: wild type X . oryzae pv. oryzae (A); xopN - xopQ - xopX - xopZ - quadruple mutant (B); T3S- mutant (C). Treated roots were subsequently stained with propidium iodide (PI) and viewed under a confocal microscope. Internalisation of PI is indicative of defense response-associated programmed cell death in rice roots. Scale bar measures 20µm. (TIF) [file pone.0075867.s002.tif]

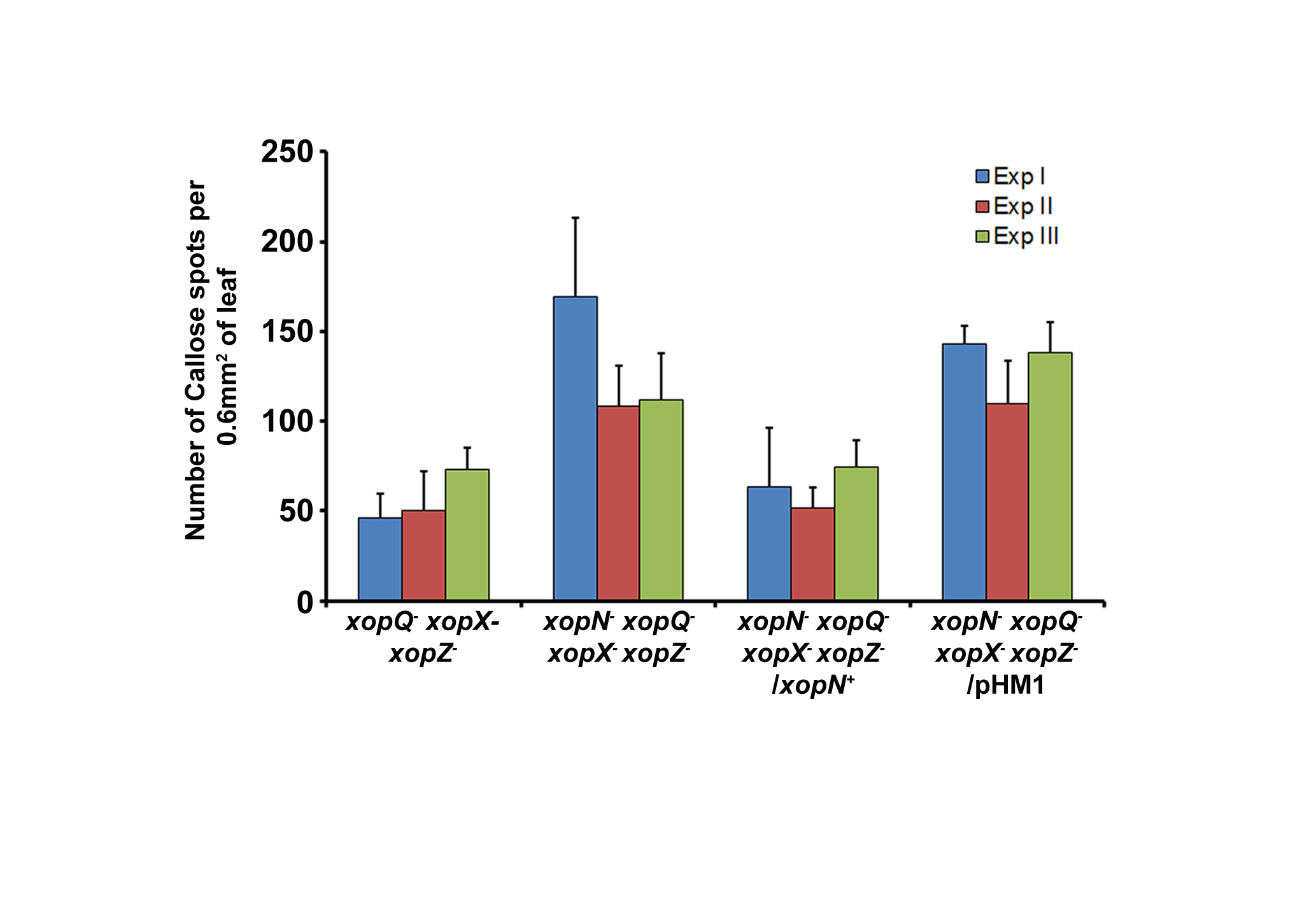

Supplement: Figure S3 — Complementation with the xopN gene reduces ability of the xopN- xopZ- xopQ- xopX- quadruple mutant to induce callose deposition in rice leaves. Rice leaves were infiltrated with one of the following: xopQ - xopX - xopZ - triple mutant, xopN - xopQ - xopX - xopZ - quadruple mutant, xopN - xopQ - xopX - xopZ - /xopN + (quadruple mutant complemented with xopN gene) and xopN - xopQ - xopX - xopZ -/pHM1 (quadruple mutant with pHM1 plasmid; vector control). The leaves were subsequently stained with aniline blue and visualized under an epifluorescence microscope. Callose deposits were quantified from 0.60 mm2 area of an infiltrated leaf. Data were collected from atleast five leaves in each experiment (three experiments indicated as ExpI, ExpII and ExpIII) and 2-3 different viewing areas from the infiltrated region of each leaf. Statistically significant differences at P < 0.05 (Student’s two-tailed t test for independent means) were obtained from leaves infiltrated with either xopN - xopQ - xopX - xopZ-/xopN + or xopQ - xopX - xopZ - triple mutant as compared to leaves treated with a xopN - xopQ - xopX - xopZ - quadruple mutant. Statistically significant differences were not observed in the following comparisons: either xopN - xopQ - xopX - xopZ-/xopN + with xopQ - xopX - xopZ - triple mutant or xopN - xopQ - xopX - xopZ - with xopN - xopZ - xopQ - xopX -/pHM1. (TIF) [file pone.0075867.s003.tif]

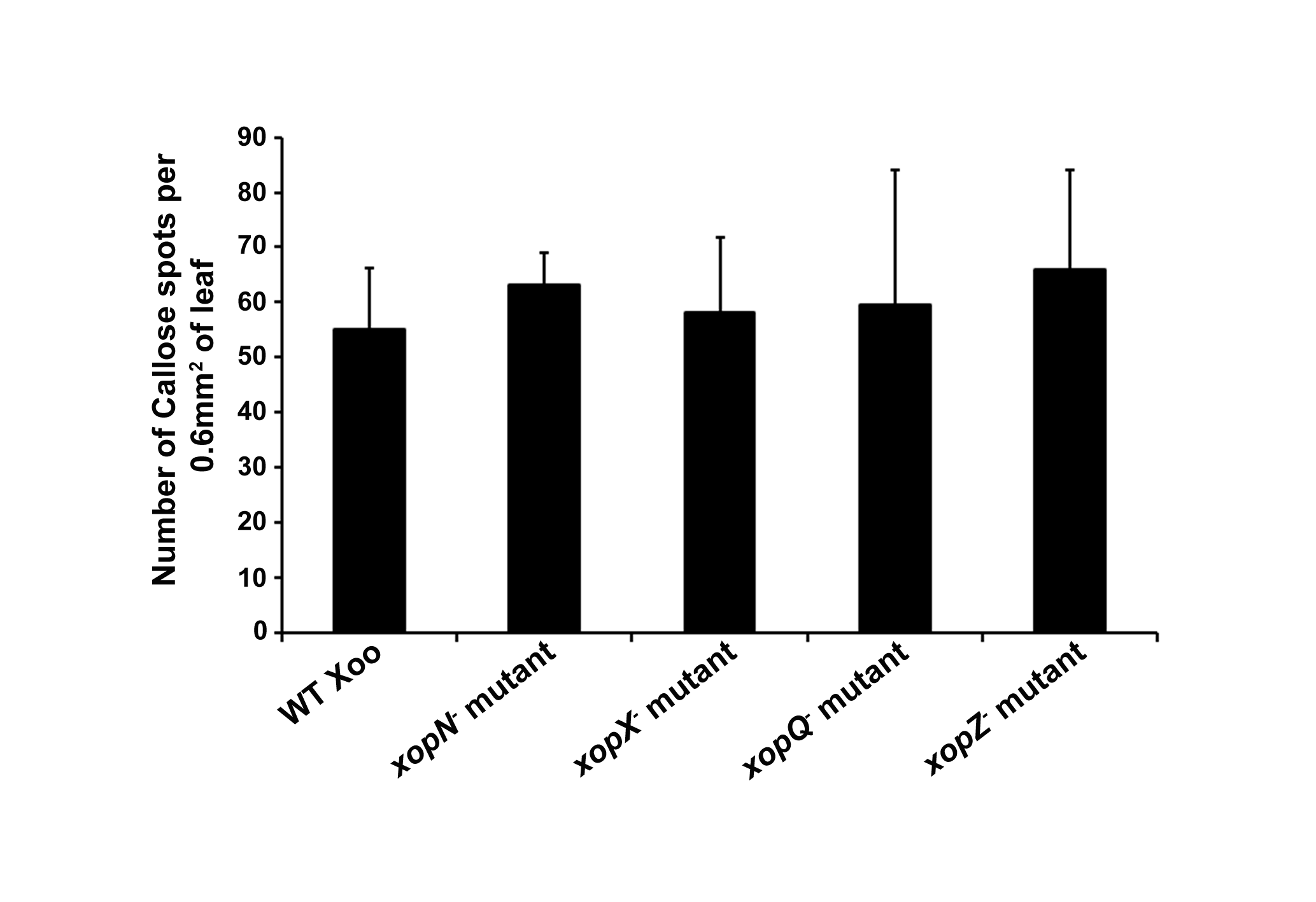

Supplement: Figure S4 — xopN-, xopQ-, xopX- or xopZ- single mutants of Xanthomonas oryzae pv. oryzae induce callose deposition at a basal level. Rice leaves were infiltrated with one of the following: wild type X . oryzae pv. oryzae , xopN - mutant, xopQ - mutant, xopX - mutant, xopZ - mutant. The leaves were subsequently stained with aniline blue and visualized under an epifluorescence microscope. Callose deposits were quantified from 0.60 mm2 area of an infiltrated leaf. Data were collected from atleast five leaves in each experiment and 2-3 different viewing areas from the infiltrated region of each leaf. Data from one experiment are represented. Similar results were obtained in independent experiments. (TIF) [file pone.0075867.s004.tif]
