# Supplementary material for: Cell Wall Degrading Enzyme Induced Rice Innate Immune Responses Are Suppressed by the Type 3 Secretion System Effectors XopN, XopQ, XopX and XopZ of Xanthomonas oryzae pv. oryzae
Source: PLoS One. 2013 Sep 26;8(9):e75867. doi: 10.1371/journal.pone.0075867 (PMC3784402; doi:10.1371/journal.pone.0075867)
Supplement: Table S1 — List of oligonucleotide primers used in this study. (DOCX) [file pone.0075867.s005.docx]

**Table S1. List of oligonucleotide primers used in this study**

| **Name** | **Nucleotide sequence (5’-3’)^a^** | **Source** |
| --- | --- | --- |
| XopPF | GTGGTTACTGTTGCCCACCA | This work |
| XopPR | TTGACGCGGTGTGCGCGCAA | This work |
| XopWF | TGGGTACCGGTGTAGTGATGAAACCGAGCCACA | This work |
| XopWR | CTGGAGCTCAATTCAACTGCCGCTACTGGAGG | This work |
| XopYF | TGGGTACCATGCGCCCTGTCCAGCCCAA | This work |
| XopYR | CTGGAGCTCCGTTAGCTCAGCCGCCGGAA | This work |
| XopAAF | TGGGTACCATGCGCGATTGCTCGTCG | This work |
| XopAAR | CTGGAGCTCACCGACCCCATACGCGTG | This work |
| XopABF | TGGGTACCGTGCCACGGCATGTGCGATG | This work |
| XopABR | CTGGAGCTCTTGCGGGGAGGGAAAGCCTC | This work |
| XopVF | CACCATGAAAATCTCCGGCTCAGC | This work |
| XopVR | TCATTCACCGTTAGGCTCAGA | This work |
| XopKF | CACCTTGGGGTTGAACGTGCTGCAGAC | This work |
| XopKR | TCAGGTCGTGGACGCAGCAGCCT | This work |
| XopFF | CACCATGAAACTCTCCGGCGGTATCGAG | This work |
| XopFR | TCATGCTCGCCCGCTTTGCCAC | This work |
| AvrBs2F | CACCATGCGTATAGGTCCTCCGCAAACG | This work |
| AvrBs2R | TCACTCCGGCTCGGTCTGGTTGG | This work |
| XopQF | CACCATGCAGCCCACCGCAATCCG | This work |
| XopQR | TCAGCGCGCATGTTCCCCCTCGT | This work |
| XopUF | CACCATGGATGCCCTGCTGCGTGCG | This work |
| XopUR | TCATGGCGCGCGCCGACGCT | This work |
| XopXF | CACCATGCTGTGGCTGCGCCTGTTCC | This work |
| XopXR | ATGCAGCGTCGAAGGACGGTCGGCTGTT | This work |
| XopTF | TGGGTACCATGGCACCAGCTCCGTGGAG | This work |
| XopTR | CTGGAGCTCTCGCCTGGACCACGTCACAAC | This work |

**Table S1. List of oligonucleotide primers used in this study (contd.)**

| **Name** | **Nucleotide sequence (5’-3’)^a^** | **Source** |
| --- | --- | --- |
| XopZF | CACCATGAGTAGTGGCGCGCCCGAACC | This work |
| XopZR | CTACGGGACTGGCTCGTAGGGAATC | This work |
| XopRF | CACCATGCGCACGAATTTTCTTCCG | This work |
| XopRR | TTATCGGTAACCGTTCTCCATTGAGTC | This work |
| XopNF | CAAGCACCAGGAGAAATGTGA | This work |
| XopNR | TTGATGCATCGCAATAGATGC | This work |
| XopXS1F | CCCAAGCTTGGGATGCTGTGGCTGCGCCTGTT | This work |
| XopXS1R | TGCTCTAGAGCATGGCCAGGCATTTGCCAGG | This work |
| XopXS3F | TGCTCTAGAGCAATTGCCTCAAAATTGTCGTTGG | This work |
| XopXS3R | CCCAAGCTTGGGTCAATGCAGCGTCGAAGGAC | This work |
| XopQS1F | CCCAAGCTTGGGCGCATGTTCCCCCTCGTC | This work |
| XopQS1R | TGCTCTAGAGCAGCCGTACCGCCAGCGTTTT | This work |
| XopQS3F | TGCTCTAGAGCAGGTCAGACGCAAGAAGCCATCG | This work |
| XopQS3R | CCCAAGCTTGGGCAGCCCACCGCAATCCGC | This work |
| XopNIF | AAGGCGCTGGAAAGCCATATC | This work |
| XopNIR | AATTTGCCCTGATCGATCTCC | This work |
| M13F | GTAAAACGACGGCCAGT | This work |
| M13R | GGAAACAGCTATGACCATG | This work |
| XopZS1F | CCCAAGCTTGGGCTGGGCATGAGTAGTGGCGCG | This work |
| XopZS1R | TGCTCTAGAGCAGCTCTGGCTGGCGATCGATAACG | This work |
| XopZS3F | TGCTCTAGAGCATCGGTGGAGATTTACATGGGAC | This work |
| XopZS3R | CCCAAGCTTGGG GGGAATCCATTGACCGACCGT | This work |
| Ubi2 | GCTCTAACCTTGAGTACCTATC | This work |
| NosT | CCCATCTCATAAATAACGTCATGC | This work |

**Table S1. List of oligonucleotide primers used in this study (contd.)**

| **Name** | **Nucleotide sequence (5’-3’)^a^** | **Source** |
| --- | --- | --- |
| XopN2F | CCCAAGCTTCAAGCACCAGGAGAAATGTGA | This work |
| XopN2R | CGAGCTCTTGATGCATCGCAATAGATGC | This work |
| XopQ2F | CCCAAGCTTGGGGGTCACCCCATGCAGCCCAC | This work |
| XopQ2R | CGAGCTCGGCCACTCCGCGAAAGCTGCC | This work |
| XopX2F | CCCAAGCTTGGGATGCTGTGGCTGCGCCTGTT | This work |
| XopX2R | CGAGCTCGGGTCAATGCAGCGTCGAAGGAC | This work |
| XopZ2F | CCCAAGCTTGGGTGGGCATGAGTAGTGGCGCG | This work |
| XopZ2R | CGAGCTCGGCACCGTTAGGATGGCACCG | This work |

^a^The underlined sequences GAGCTC, AAGCTT and TCTAGA indicate engineered restriction sites for *Sac*I, *Hind*III and *Xba*I respectively, in primers
