# Supplementary material for: Cell Wall Degrading Enzyme Induced Rice Innate Immune Responses Are Suppressed by the Type 3 Secretion System Effectors XopN, XopQ, XopX and XopZ of Xanthomonas oryzae pv. oryzae
Source: PLoS One. 2013 Sep 26;8(9):e75867. doi: 10.1371/journal.pone.0075867 (PMC3784402; doi:10.1371/journal.pone.0075867)
Supplement: Table S2 — List of oligonucleotide primers used for RT-PCR. (DOCX) [file pone.0075867.s006.docx]

**Table S2. List of oligonucleotide primers used for RT-PCR**

| **Gene name** | **Primer name** | **Primer sequence (5’ to 3’)** | **Source** |
| --- | --- | --- | --- |
| *xopN* | XopNRTF | ACGCATTGGCCAGATGTTTC | This work |
| *xopN* | XopNRTR | ATTGCCAATGCCTGTGGGAT | This work |
| *xopP* | XopPRTF | AACGTCACCATCTCGACCCTTTG | This work |
| *xopP* | XopPRTR | AGAATTTCCTGGTGCCGCAGTT | This work |
| *xopQ* | XopQRTF | ATTCCTCTGCGCATCCTGACCAA | This work |
| *xopQ* | XopQRTR | TGCCTTCCCACAGGCCTTTCAA | This work |
| *xopR* | XopRRTF | TGCTGTTGCCATCTGTCTTCGT | This work |
| *xopR* | XopRRTR | ATGGGCAAACCCTTCTACCTGA | This work |
| *xopX* | XopXRTF | GCACCAATTGGGCTTCACCAACTA | This work |
| *xopX* | XopXRTR | GGCGCACCTGTTGAAATGCATC | This work |
| *xopZ* | XopZRTF | AACGCACTCAAGCTGGGAATGAAG | This work |
| *xopZ* | XopZRTR | AACTGCCCGCACGTCATTTGAA | This work |
| *GAPDH* | GAPDHF | AGTGGGGTTACAGCAACCGT | This work |
| *GAPDH* | GAPDHR | TCCTGATGAACACCGTCCAC | This work |
